# Fractional model for Middle East respiratory syndrome coronavirus on a complex heterogeneous network

**DOI:** 10.1038/s41598-022-24814-1

**Published:** 2022-12-01

**Authors:** H. A. A. El-Saka, Ibrahim Obaya, Seyeon Lee, Bongsoo Jang

**Affiliations:** 1 Mathematics Department, Faculty of Science, Damietta University, New Damietta, 34517 Egypt; 2 Basic Science Department, Nile Higher Institute for Engineering and Technology, Mansoura, Egypt; 3 Department of Mathematics, Faculty of Science, Mansoura University, P.O. Box 64, Mansoura, 35516 Egypt; 4 Division of Industrial Mathematics, National Institute for Mathematical Sciences, Daejeon, 34047 Republic of Korea; 5 Department of Mathematical Sciences, Ulsan National Institute of Science and Technology (UNIST), Ulsan, 44919 Republic of Korea

**Keywords:** Diseases, Mathematics and computing

## Abstract

In this paper, we present a new fractional epidemiological model on a heterogeneous network to investigate Middle East respiratory syndrome (MERS-CoV), which is caused by a virus in the coronavirus family. We also consider the development of equations for the camel population, given that it is the primary animal source of the virus, as well as direct human interaction with this population. The model is configured in an SIS form for both the human population and the camel population. We study the equilibrium positions of the system and the conditions for the existence of each of them, as well as the local stability of each equilibrium position. Then, we provide some numerical examples that compare real data and numerical results.

## Introduction

Several dynamic systems have been developed to study the epidemiological spread of many infectious diseases. Epidemiological systems are among the major tools for studying and developing epidemiological dynamics. Researchers aim to make epidemiological models more realistic so that they are more accurate in describing the spread of an epidemic. Among the mathematical tools used in developing epidemiological models are fractional calculus and network science. In this context, we have developed a model that examines the Middle East respiratory syndrome coronavirus (MERS-CoV), which in turn helps us to improve our understanding of the spread of this disease.

MERS-CoV is one of the most dangerous viruses that has emerged in the last seven years. The number of deaths among individuals infected with the virus has reached approximately 35 percent. MERS-CoV is classified as a zoonotic virus, in which an animal is the carrier of the virus and the source of its spread. Camels are the main reservoir hosts of MERS-CoV and help it spread. This is due to the presence of antibodies for MERS-CoV in some caravans of camels^1^.

The Arabian peninsula has one of the largest camel populations in the world. Therefore, we find that the places with the most MERS-CoV infections are in the Arabian Gulf region, which primarily includes Saudi Arabia. Saudi Arabia has the highest rate of infection in the Gulf region, almost 80 percent of total cases. In addition, we find that the population of the Arabian Gulf region deals with camels closely and continuously, and camels in these areas are considered a national resource. Consuming camel meat and milk is common in the Gulf region. Camel urine is also used in some therapeutic practices by some residents, and breeders come into close contact with camels^2,3^.

On the other hand, MERS-CoV is only transmitted from person to person in cases of close contact with an infected person. Therefore, in making the model, both person-to-person and camel-to-person transitions were considered. There is also no evidence that the virus has passed from an infected person to an uninfected camel thus far^4,5^.

The spread of the infection is not limited to the Gulf region; it has spread to 27 countries worldwide. Therefore, we consider in this case that the virus is spread through a network where the nodes represent the elements of the community of humans and camels and the links in the network represent communication between elements of the community. The representation of this community with a heterogeneous network plays an important role in describing the pattern of communication between the elements of the community as well as the mechanism of the spread of the disease. Several epidemiological models have been presented in the form of heterogeneous networks and calculations of the basic reproductive number, which in turn determine the spread threshold in the epidemic network^6^.

Fractional epidemiological modeling is one of the most important tools used to develop the study of epidemiological systems and obtain more realistic results. The use of fractional models is not limited to epidemiological models but has been extended to many fields of research in engineering and economics. The use of mathematical models in a fractional form is important because the definition of a fractional derivative includes a representation of memory as well as the effect of nonlocality.

Several mathematical models have been introduced to describe the dynamics of infectious diseases from an epidemiological perspective^7–16,25–27^.

Many works and mathematical models have used a heterogeneous network pattern in simulating an epidemiological community, and this new configuration of the model needs more sophisticated calculation methods. Liu et al.^21^ presented a simple SIS model for a fixed-number population in which they showed how to calculate the equilibrium positions and determine the stability of each equilibrium position.

There are more sophisticated models that focus on certain phenomena. Soovoogeet et al.^26^ considered the rate of recovery from infection as a function of the number of infected patients. This function shows the effectiveness of the treatment on the infected person and its effect on the spread of infection. However, this model is not properly created in a heterogeneous network pattern. See also^27^.

Liu et al.^28^, who studied the SIS model in a heterogeneous network, used a nonlinear incidence rate that expresses the desire of infected persons to be more careful to avoid infection. On this basis, we have created our model. This will be explained in detail in “Model formulation” section. Additionally, some fractional models have been formed that account for quarantine classes and vaccination effects. A good discussion of the threshold of proposed stochastic models with slight or severe noise is given in^29–34^.

This combination of fractional differentiation and heterogeneous networks has been found to produce more accurate and realistic results. It also provides broader conditions for stabilizing the model equilibrium points as well as disease transmission thresholds, which are linked to the network topology. Some epidemiological models have also been presented to study the interaction between the host community and the vector community^24–28^.

In our model, the community where the virus spreads includes both humans and camels. When a susceptible individual is exposed to infection, it is possible that he or she may become infected again after recovering, so we chose the SIS pattern to describe the transition between humans. The same pattern of SIS is also used to describe the transition between camels, where the infection causes a moderate condition in the infected camel^12^.

Direct contact with camels is one of the most important reasons for the transmission of the disease from infected camels to noninfected individuals, such as in direct contact by individuals who breed and trade camels, those who supervise them for competitions and those working on farms that breed camels. Therefore, we will study the impact of adherence to preventive measures while dealing with camels.

In this paper, we present the preliminaries of fractional calculus in “Preliminaries” section. In “Model formulation” section, we present an epidemiological model in fractional form to study the spread of MERS-CoV in a heterogeneous network containing both humans and camels. In “Model analysis” section, we calculate the equilibrium positions and the basic reproductive number. In “Numerical simulation” section, we study the stability of these equilibrium positions. Finally, a numerical simulation is presented.

## Preliminaries

We introduce some basic definitions of fractional calculus as follows:

### Definition 1

The operator ${}_{a}{I}_{t}^{\alpha }$ is called the fractional integral of order $\alpha$, where $\alpha \in (0,\infty )$ (the Riemann–Liouville fractional integral) is defined as:

$${}_{a}{I}_{t}^{\alpha }f\left(t\right)=\frac{1}{\Gamma \left(\alpha \right)}{\int }_{a}^{t}{\left(t-s\right)}^{\alpha -1}f\left(s\right)ds.$$

### Definition 2

The operator ${}_{a}^{RL}{D}_{t}^{\alpha }$ is called the Riemann–Liouville fractional derivative of order $\alpha$ and is defined as:

$${}_{a}^{RL}{D}_{t}^{\alpha }f\left(t\right)=\frac{1}{\Gamma \left(n-\alpha \right)}\frac{{d}^{n}}{d{t}^{n}}{\int }_{a}^{t}{\left(t-s\right)}^{n-\alpha -1}f\left(s\right)ds,$$

where $n$ is a positive integer and $n-1<\alpha <n$.

### Definition 3

The operator ${}_{a}^{C}{D}_{t}^{\alpha }$ is called the Caputo fractional derivative of order $\alpha$ and is defined as:

$${}_{a}^{C}{D}_{t}^{\alpha }f\left(t\right)=\frac{1}{\Gamma (n-\alpha )}{\int }_{a}^{t}{\left(t-s\right)}^{n-\alpha -1}{f}^{\left(n\right)}\left(s\right)ds,$$

where $n$ is a positive integer and $n-1<\alpha \le n$.

In the special case when $0<\alpha \le 1$, the Caputo fractional derivative is:

$${}_{a}^{C}{D}_{t}^{\alpha }f\left(t\right)=\frac{1}{\Gamma (1-\alpha )}{\int }_{a}^{t}{\left(t-s\right)}^{-\alpha }{f}^{^{\prime}}\left(s\right)ds.$$

Several properties of fractional calculus can be found in^7,8,19,20,22,23^.

## Model formulation

In this model, it is known that the transmission of MERS-CoV depends on camels as an animal source of the virus. Therefore, our population contains both camels as an infection vector and humans as a host. The SIS pattern is used to describe the transmission route. The infection spreads due to contact between humans and contact between humans and camels. A camel can transmit the infection to individuals who are close to it and who deal with it continuously. This means that there is a selectivity pattern during the transmission of the infection that makes the disease spread in a heterogeneous way between camels and humans. The network nodes represent both humans and camels, and the links represent daily contacts. Human nodes can have a susceptible status or infected status, similarly to camel nodes. At any point in time, the process of infection transmission can be described as follows: susceptible individuals can be infected during contact with infected individuals. Infected individuals become susceptible again after recovering. Susceptible individuals can be infected while dealing with infected camels or infected humans and become infected. Susceptible camels can be infected during contact with infected camels, which become susceptible again. Let ${S}^{h}(t)$ and ${I}^{h}(t)$ denote the proportion of susceptible humans and the proportion of infected humans, respectively. Let ${S}^{c}(t)$ and ${I}^{c}(t)$ denote the proportion of susceptible camels and the proportion of infected camels, respectively. Suppose that the human network has the composed degree distribution $P(k,m)$, which gives the ratio of nodes that have $k$ links with humans and $m$ links with camels. Therefore, the nodes in each class have two degrees, $k$ and $m$. Similarly, the camel network has the composed degree distribution $P(l,m)$, which gives the ratio of nodes that have $m$ links with humans and $l$ links with camels. The degree distributions can be defined as $P\left(i,j\right)=P\left(i\right)*P(j)$ for any $i,j$. Let ${S}_{\left(k,m\right)}^{h}(t)$ and ${I}_{\left(k,m\right)}^{h}(t)$ be the proportions of susceptible and infected host nodes of degrees $k$ and $m$ at time $t$, where the pair $\left(k,m\right)$ belongs to a nonempty finite set ${\Omega }_{1}$,

$${\Omega }_{1}=\left\{\left(k,m\right): {k}_{min}\le k\le {k}_{max},{m}_{min}\le m\le {m}_{max}\right\}.$$

Similar to the host nodes, let ${S}_{\left(l,m\right)}^{c}(t)$ and ${I}_{\left(l,m\right)}^{c}(t)$ be the proportions of susceptible and infected vector nodes of degrees $m$ and $l$ at time $t$, where the pair $\left(l,m\right)$ belongs to a nonempty finite set ${\Omega }_{2}$,

$${\Omega }_{2}=\left\{\left(l,m\right): {l}_{min}\le l\le {l}_{max},{m}_{min}\le m\le {m}_{max}\right\}.$$

We suppose that ${k}_{max}={l}_{max}={m}_{max}={\mathbb{N}}$ and ${k}_{min}={l}_{min}={m}_{min}=1$.

Let ${b}_{1}$ be the birth and death rate in the host individuals and ${b}_{2}$ be the birth and death rate in the vector population.

**Figure 1:**
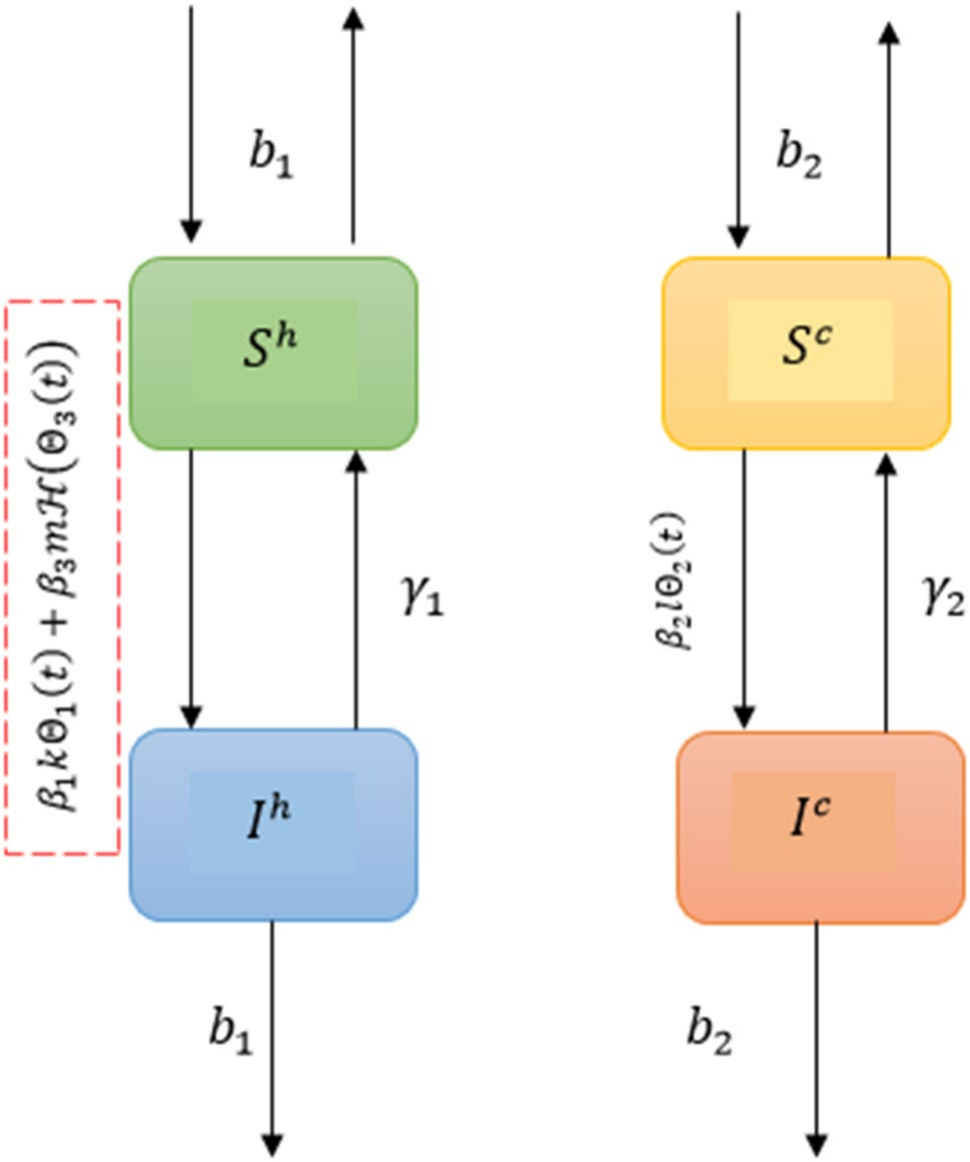
Dynamics diagram of Model (2).

According to the above dynamic description of the system (see Fig. 1), the fractional model in Caputo form is defined as:

1 $$\begin{aligned} & {}_{0}^{C} D_{t}^{\alpha } S_{{\left( {k,m} \right)}}^{h} \left( t \right) = b_{1} - b_{1} S_{{\left( {k,m} \right)}}^{h} \left( t \right) - \beta_{1} kS_{{\left( {k,m} \right)}}^{h} \left( t \right){\Theta }_{1} \left( t \right) - \beta_{3} mS_{{\left( {k,m} \right)}}^{h} \left( t \right){\Theta }_{3} \left( t \right) + \gamma_{1} I_{{\left( {k,m} \right)}}^{h} \left( t \right), \\ & {}_{0}^{C} D_{t}^{\alpha } I_{{\left( {k,m} \right)}}^{h} \left( t \right) = \beta_{1} kS_{{\left( {k,m} \right)}}^{h} \left( t \right){\Theta }_{1} \left( t \right) + \beta_{3} mS_{{\left( {k,m} \right)}}^{h} \left( t \right){\Theta }_{3} \left( t \right) - \gamma_{1} I_{{\left( {k,m} \right)}}^{h} \left( t \right) - b_{1} I_{{\left( {k,m} \right)}}^{h} \left( t \right), \\ & {}_{0}^{C} D_{t}^{\alpha } S_{{\left( {l,m} \right)}}^{c} \left( t \right) = b_{2} - b_{2} S_{{\left( {l,m} \right)}}^{c} \left( t \right) - \beta_{2} lS_{{\left( {l,m} \right)}}^{c} \left( t \right){\Theta }_{2} \left( t \right) + \gamma_{2} I_{{\left( {l,m} \right)}}^{c} \left( t \right), \\ & {}_{0}^{C} D_{t}^{\alpha } I_{{\left( {l,m} \right)}}^{c} \left( t \right) = \beta_{2} lS_{{\left( {l,m} \right)}}^{c} \left( t \right){\Theta }_{2} \left( t \right) - \gamma_{2} I_{{\left( {l,m} \right)}}^{c} \left( t \right) - b_{2} I_{{\left( {l,m} \right)}}^{c} \left( t \right), \\ \end{aligned}$$

where $$0<\alpha \le 1$$ and

$${\Theta }_{1}\left(t\right)=\frac{1}{\langle k\rangle }\sum_{m=1}^{\mathbb{N}}\sum_{k=1}^{\mathbb{N}}kP\left(k,m\right){I}_{\left(k,m\right)}^{h}\left(t\right),$$

which represents the probability of a given link being connected with an infected individual, where $\langle k\rangle =\sum_{m=1}^{\mathbb{N}}\sum_{k=1}^{\mathbb{N}}kP(k,m)$ is the average degree of the disease in the human nodes. In addition,

$${\Theta }_{2}\left(t\right)=\frac{1}{\langle l\rangle }\sum_{m=1}^{\mathbb{N}}\sum_{l=1}^{\mathbb{N}}lP(l,m) {I}_{\left(l,m\right)}^{c}(t),$$

which represents the probability of a given link being connected with an infected camel, where $\langle l\rangle =\sum_{m=1}^{\mathbb{N}}\sum_{l=1}^{\mathbb{N}}l P(l,m)$ is the average degree of the disease in the camel nodes. Additionally, we define ${\Theta }_{3}\left(t\right)$ as

$${\Theta }_{3}\left(t\right)=\frac{1}{\langle m\rangle }\sum_{m=1}^{\mathbb{N}}\sum_{l=1}^{\mathbb{N}}mP(l,m) {I}_{\left(l,m\right)}^{c}(t).$$

This describes the probability of a given link between a human and an infected camel, where $\langle m\rangle =\sum_{m=1}^{\mathbb{N}}\sum_{l=1}^{\mathbb{N}}mP(l,m)$ is the average degree of the disease between human and camel nodes.

A description of the parameters is given in Table 1. At any time $t$, the fraction of human nodes with the same degrees $k$ and $m$, ${S}_{\left(k,m\right)}^{h}\left(t\right)+{I}_{\left(k,m\right)}^{h}\left(t\right)=1$, is constant. Similarly, for the camel nodes, ${S}_{\left(l,m\right)}^{c}\left(t\right)+{I}_{\left(l,m\right)}^{c}\left(t\right)=1$.

**Table 1:** Parameter descriptions.

Parameter	Description
${\beta }_{1}$	The transmission rate from an infected human to a susceptible human
${\beta }_{2}$	The transmission rate from an infected camel to a susceptible camel
${\beta }_{3}$	The transmission rate from an infected camel to a susceptible human
${b}_{1}$	Birth and death rate in the human population
${b}_{2}$	Birth and death rate in the camel population
${\gamma }_{1}$	Recovery rate of an infected host (human), who then becomes susceptible
${\gamma }_{2}$	Recovery rate of an infected vector (camel), which then becomes susceptible
$\omega$	Rate of commitment to preventive measures

Due to the importance of individuals who deal closely with camels being committed to following preventive instructions that help to reduce the spread of the disease, the term ${\beta }_{3}m{S}_{\left(k,m\right)}^{h}\left(t\right){\Theta }_{3}\left(t\right)$, which represents the influence of infected camels on uninfected individuals, can be modified to ${\beta }_{3}m{S}_{\left(k,m\right)}^{h}\left(t\right)\mathcal{H}({\Theta }_{3}\left(t\right))$, where $\mathcal{H}\left({\Theta }_{3}\left(t\right)\right)={\Theta }_{3}\left(t\right){e}^{-\omega {\Theta }_{3}\left(t\right)}$. The new parameter $\omega$ reflects the rate of taking preventive measures by susceptible individuals who are in close contact with infected camels, where $\omega \in [\mathrm{0,1}]$. A small $\omega$ value means that few susceptible individuals comply with preventive instructions. In contrast, a large $\omega$ means that almost all susceptible individuals adhere to preventive instructions. When $\omega$ is equal to zero, this means none of the susceptible individuals complies with preventive instructions, and the function $\mathcal{H}\left({\Theta }_{3}\left(t\right)\right)$ becomes ${\Theta }_{3}\left(t\right)$.

Thus, the fractional model in (1) can be written as

2 $$\begin{aligned} & {}_{0}^{C} D_{t}^{\alpha } S_{{\left( {k,m} \right)}}^{h} \left( t \right) = b_{1} - b_{1} S_{{\left( {k,m} \right)}}^{h} \left( t \right) - \beta_{1} kS_{{\left( {k,m} \right)}}^{h} \left( t \right){\Theta }_{1} \left( t \right) - \beta_{3} mS_{{\left( {k,m} \right)}}^{h} \left( t \right){\Theta }_{3} \left( t \right)e^{{ - \omega {\Theta }_{3} \left( t \right)}} + \gamma_{1} I_{{\left( {k,m} \right)}}^{h} \left( t \right), \\ & {}_{0}^{C} D_{t}^{\alpha } I_{{\left( {k,m} \right)}}^{h} \left( t \right) = \beta_{1} kS_{{\left( {k,m} \right)}}^{h} \left( t \right){\Theta }_{1} \left( t \right) + \beta_{3} mS_{{\left( {k,m} \right)}}^{h} \left( t \right){\Theta }_{3} \left( t \right)e^{{ - \omega {\Theta }_{3} \left( t \right)}} - \left( {b_{1} + \gamma_{1} } \right)I_{{\left( {k,m} \right)}}^{h} \left( t \right), \\ & {}_{0}^{C} D_{t}^{\alpha } S_{{\left( {l,m} \right)}}^{c} \left( t \right) = b_{2} - b_{2} S_{{\left( {l,m} \right)}}^{c} \left( t \right) - \beta_{2} lS_{{\left( {l,m} \right)}}^{c} \left( t \right){\Theta }_{2} \left( t \right) + \gamma_{2} I_{{\left( {l,m} \right)}}^{c} \left( t \right), \\ & {}_{0}^{C} D_{t}^{\alpha } I_{{\left( {l,m} \right)}}^{c} \left( t \right) = \beta_{2} lS_{{\left( {l,m} \right)}}^{c} \left( t \right){\Theta }_{2} \left( t \right) - \left( {b_{2} + \gamma_{2} } \right)I_{{\left( {l,m} \right)}}^{c} \left( t \right). \\ \end{aligned}$$

Let

$$\mathfrak{J}=\left\{\left.\left({S}_{\left(k,m\right)}^{h}\left(t\right),{I}_{\left(k,m\right)}^{h}\left(t\right),{S}_{\left(l,m\right)}^{c}\left(t\right),{I}_{\left(l,m\right)}^{c}(t)\right)\in {R}_{+}^{4k},{k}_{min}\le k\le {k}_{max},{m}_{min}\le m\le {m}_{max},{l}_{min}\le l\le {l}_{max}\right|{S}_{\left(k,m\right)}^{h}\left(t\right)+{I}_{\left(k,m\right)}^{h}\left(t\right)=1, {S}_{\left(l,m\right)}^{c}\left(t\right)+{I}_{\left(l,m\right)}^{c}\left(t\right)=1\right\}$$

be a closed positive invariant set for system (2).

The analysis of Model (2) is given in the following section. Several mathematical models of fractional order used in analyzing the dynamics of epidemiological diseases have been investigated in^7–16,25–27^.

## Model analysis

### Equilibrium points

#### Theorem 1

(i) If ${\mathcal{R}}_{0}^{c}<1$ and ${\mathcal{R}}_{0}^{h}<1$, then we have only the disease-free equilibrium point.
  $${E}_{0}={\left\{\mathrm{1,0},\mathrm{1,0}\right\}}_{\begin{array}{c}\forall \left(k,m\right) \in {\Omega }_{1}\\ \forall \left(l,m\right) \in {\Omega }_{2}\end{array}},$$
  where
  3 $${\mathcal{R}}_{0}^{c} = \frac{{\langle {l}^{2}\rangle}}{\langle {l}\rangle}\frac{{\beta_{2} }}{{\left( {\gamma_{2} + b_{2} } \right)}},$$
  4 $${\mathcal{R}}_{0}^{h} = \frac{{\langle {k}^{2}\rangle }}{\langle {k}\rangle}\frac{{\beta_{1} }}{{\left( {\gamma_{1} + b_{1} } \right)}},$$
  $$\langle {l}^{2}\rangle = \mathop \sum \limits_{m = 1}^{{\mathbb{N}}} \mathop \sum \limits_{l = 1}^{{\mathbb{N}}} l^{2} P\left( {l,m} \right),$$
  and
  $$\langle {k}^{2}\rangle =\sum_{m=1}^{\mathbb{N}}\sum_{k=1}^{\mathbb{N}}{k}^{2}P\left(k,m\right).$$
(ii) If ${\mathcal{R}}_{0}^{c}>1$ and ${\widehat{\mathcal{R}}}_{0}^{h}>1$, then ${E}_{0}$ exists in addition to an endemic equilibrium point
  $${E}_{1}={\left\{{S}_{\left(k,m\right)}^{{h}^{*}},{I}_{\left(k,m\right)}^{{h}^{*}},{S}_{\left(l,m\right)}^{{c}^{*}},{I}_{\left(l,m\right)}^{{c}^{*}}\right\}}_{\begin{array}{c}\forall \left(k,m\right) \in {\Omega }_{1}\\ \forall \left(l,m\right) \in {\Omega }_{2}\end{array}}.$$
  where
  $${\widehat{\mathcal{R}}}_{0}^{h}=\frac{1}{\langle k\rangle }\sum_{m=1}^{\mathbb{N}}\sum_{k=1}^{\mathbb{N}}{k}^{2}P(k,m) \frac{{\beta }_{1}\left({\gamma }_{1}+{b}_{1}\right)}{{\left({\beta }_{3}m{\Theta }_{3}\left(t\right){e}^{-\omega {\Theta }_{3}\left(t\right)}+{\gamma }_{1}+{b}_{1}\right)}^{2}}.$$
(iii) If ${\mathcal{R}}_{0}^{c}<1$ and ${\mathcal{R}}_{0}^{h}>1$, then ${E}_{0}$ exists in addition to a human endemic equilibrium point
  $${E}_{2}={\left\{{S}_{\left(k,m\right)}^{{h}^{**}},{I}_{\left(k,m\right)}^{{h}^{**}},\mathrm{1,0}\right\}}_{\begin{array}{c}\forall \left(k,m\right) \in {\Omega }_{1}\\ \forall \left(l,m\right) \in {\Omega }_{2}\end{array}}.$$
  See the detailed proof of Theorem 1 in the Supplementary material (Appendix A).

#### Remark 1

The previous equilibrium positions reflect the normal pattern of spread of the animal virus. We find that the first situation describes the state of society without an epidemic. The second situation shows that the spread of the virus in the animal community will in turn lead to spread in the human community, and the community will be infected with the virus completely. The third situation shows that it is possible for the animal community to be free of the virus while it remains common among people, but this situation does not occur until after it has spread in the animal community first.

### The basic reproductive number

In performing the next-generation method^17,18,24^, we will focus on two equations of Model (2), which represent two compartments ${I}_{\left(k,m\right)}^{h}$ and ${I}_{\left(l,m\right)}^{c}$:

5 $$\begin{aligned} & {}_{0}^{C} D_{t}^{\alpha } I_{{\left( {k,m} \right)}}^{h} \left( t \right) = \beta_{1} kS_{{\left( {k,m} \right)}}^{h} \left( t \right){\Theta }_{1} \left( t \right) + \beta_{3} mS_{{\left( {k,m} \right)}}^{h} \left( t \right){\Theta }_{3} \left( t \right)e^{{ - \omega {\Theta }_{3} \left( t \right)}} - \left( {\gamma_{1} + b_{1} } \right)I_{{\left( {k,m} \right)}}^{h} \left( t \right), \\ & {}_{0}^{C} D_{t}^{\alpha } I_{{\left( {l,m} \right)}}^{c} \left( t \right) = \beta_{2} lS_{{\left( {l,m} \right)}}^{c} \left( t \right){\Theta }_{2} \left( t \right) - \left( {\gamma_{2} + b_{2} } \right)I_{{\left( {l,m} \right)}}^{c} \left( t \right). \\ \end{aligned}$$

The rate of new infected nodes entering the two compartments ${I}_{\left(k,m\right)}^{h}$ and ${I}_{\left(l,m\right)}^{c}$ is represented by the matrix $F$:

6 $$F={\left(\begin{array}{cc}{\mathcal{F}}_{11}& {\mathcal{F}}_{12}\\ {\mathcal{F}}_{21}& {\mathcal{F}}_{22}\end{array}\right)}_{2{\mathbb{N}}^{2}\times 2{\mathbb{N}}^{2}},$$

where ${\mathcal{F}}_{11},{\mathcal{F}}_{12},{\mathcal{F}}_{21}$ and ${\mathcal{F}}_{22}$ are ${\mathbb{N}}^{2}\times {\mathbb{N}}^{2}$ matrices given by

$${\mathcal{F}}_{11}=\frac{{\beta }_{1}}{\langle k\rangle }{\left(\begin{array}{ccc}\begin{array}{ccc}P\left(\mathrm{1,1}\right)& \cdots & P\left(1,{\mathbb{N}}\right)\\ \vdots & \ddots & \vdots \\ P\left(\mathrm{1,1}\right)& \cdots & P\left(1,{\mathbb{N}}\right)\end{array}& \begin{array}{ccc}2P\left(\mathrm{2,1}\right)& \cdots & 2P\left(2,{\mathbb{N}}\right)\\ \vdots & \ddots & \vdots \\ 2P\left(\mathrm{2,1}\right)& \cdots & 2P\left(2,{\mathbb{N}}\right)\end{array}& \begin{array}{cc}\dots & \begin{array}{ccc}{\mathbb{N}}P\left({\mathbb{N}},1\right)& \cdots & {\mathbb{N}}P\left({\mathbb{N}},{\mathbb{N}}\right)\\ \vdots & \ddots & \vdots \\ {\mathbb{N}}P\left({\mathbb{N}},1\right)& \cdots & {\mathbb{N}}P\left({\mathbb{N}},{\mathbb{N}}\right)\end{array}\end{array}\\ \begin{array}{ccc}2P\left(\mathrm{1,1}\right)& \cdots & 2P\left(1,{\mathbb{N}}\right)\\ \vdots & \ddots & \vdots \\ 2P\left(\mathrm{1,1}\right)& \cdots & 2P\left(1,{\mathbb{N}}\right)\end{array}& \begin{array}{ccc}{2}^{2}P\left(\mathrm{2,1}\right)& \cdots & {2}^{2}P\left(2,{\mathbb{N}}\right)\\ \vdots & \ddots & \vdots \\ {2}^{2}P\left(\mathrm{2,1}\right)& \cdots & {2}^{2}P\left(2,{\mathbb{N}}\right)\end{array}& \begin{array}{cc}\dots & \begin{array}{ccc}2{\mathbb{N}}P\left({\mathbb{N}},1\right)& \cdots & 2{\mathbb{N}}P\left({\mathbb{N}},{\mathbb{N}}\right)\\ \vdots & \ddots & \vdots \\ 2{\mathbb{N}}P\left({\mathbb{N}},1\right)& \cdots & 2{\mathbb{N}}P\left({\mathbb{N}},{\mathbb{N}}\right)\end{array}\end{array}\\ \begin{array}{c}\vdots \\ \begin{array}{ccc}{\mathbb{N}}P\left(\mathrm{1,1}\right)& \cdots & {\mathbb{N}}P\left(1,{\mathbb{N}}\right)\\ \vdots & \ddots & \vdots \\ {\mathbb{N}}P\left(\mathrm{1,1}\right)& \cdots & {\mathbb{N}}P\left(1,{\mathbb{N}}\right)\end{array}\end{array}& \begin{array}{c}\vdots \\ \begin{array}{ccc}2{\mathbb{N}}P\left(\mathrm{2,1}\right)& \cdots & 2{\mathbb{N}}P\left(2,{\mathbb{N}}\right)\\ \vdots & \ddots & \vdots \\ 2{\mathbb{N}}P\left(\mathrm{2,1}\right)& \cdots & 2{\mathbb{N}}P\left(2,{\mathbb{N}}\right)\end{array}\end{array}& \begin{array}{cc}\begin{array}{c}\ddots \\ \dots \end{array}& \begin{array}{c}\vdots \\ \begin{array}{ccc}{\mathbb{N}}^{2}P\left({\mathbb{N}},1\right)& \cdots & {\mathbb{N}}^{2}P\left({\mathbb{N}},{\mathbb{N}}\right)\\ \vdots & \ddots & \vdots \\ {\mathbb{N}}^{2}P\left({\mathbb{N}},1\right)& \cdots & {\mathbb{N}}^{2}P\left({\mathbb{N}},{\mathbb{N}}\right)\end{array}\end{array}\end{array}\end{array}\right)}_{{\mathbb{N}}^{2}\times {\mathbb{N}}^{2}},$$

$${\mathcal{F}}_{12}=\frac{{\beta }_{3}}{\langle m\rangle }{\left(\begin{array}{ccc}\begin{array}{ccc}P\left(\mathrm{1,1}\right)& \cdots & P\left(1,{\mathbb{N}}\right)\\ \vdots & \ddots & \vdots \\ P\left(\mathrm{1,1}\right)& \cdots & P\left(1,{\mathbb{N}}\right)\end{array}& \begin{array}{ccc}2P\left(\mathrm{2,1}\right)& \cdots & 2P\left(2,{\mathbb{N}}\right)\\ \vdots & \ddots & \vdots \\ 2P\left(\mathrm{2,1}\right)& \cdots & 2P\left(2,{\mathbb{N}}\right)\end{array}& \begin{array}{cc}\dots & \begin{array}{ccc}{\mathbb{N}}P\left({\mathbb{N}},1\right)& \cdots & {\mathbb{N}}P\left({\mathbb{N}},{\mathbb{N}}\right)\\ \vdots & \ddots & \vdots \\ {\mathbb{N}}P\left({\mathbb{N}},1\right)& \cdots & {\mathbb{N}}P\left({\mathbb{N}},{\mathbb{N}}\right)\end{array}\end{array}\\ \begin{array}{ccc}2P\left(\mathrm{1,1}\right)& \cdots & 2P\left(1,{\mathbb{N}}\right)\\ \vdots & \ddots & \vdots \\ 2P\left(\mathrm{1,1}\right)& \cdots & 2P\left(1,{\mathbb{N}}\right)\end{array}& \begin{array}{ccc}{2}^{2}P\left(\mathrm{2,1}\right)& \cdots & {2}^{2}P\left(2,{\mathbb{N}}\right)\\ \vdots & \ddots & \vdots \\ {2}^{2}P\left(\mathrm{2,1}\right)& \cdots & {2}^{2}P\left(2,{\mathbb{N}}\right)\end{array}& \begin{array}{cc}\dots & \begin{array}{ccc}2{\mathbb{N}}P\left({\mathbb{N}},1\right)& \cdots & 2{\mathbb{N}}P\left({\mathbb{N}},{\mathbb{N}}\right)\\ \vdots & \ddots & \vdots \\ 2{\mathbb{N}}P\left({\mathbb{N}},1\right)& \cdots & 2{\mathbb{N}}P\left({\mathbb{N}},{\mathbb{N}}\right)\end{array}\end{array}\\ \begin{array}{c}\vdots \\ \begin{array}{ccc}{\mathbb{N}}P\left(\mathrm{1,1}\right)& \cdots & {\mathbb{N}}P\left(1,{\mathbb{N}}\right)\\ \vdots & \ddots & \vdots \\ {\mathbb{N}}P\left(\mathrm{1,1}\right)& \cdots & {\mathbb{N}}P\left(1,{\mathbb{N}}\right)\end{array}\end{array}& \begin{array}{c}\vdots \\ \begin{array}{ccc}2{\mathbb{N}}P\left(\mathrm{2,1}\right)& \cdots & 2{\mathbb{N}}P\left(2,{\mathbb{N}}\right)\\ \vdots & \ddots & \vdots \\ 2{\mathbb{N}}P\left(\mathrm{2,1}\right)& \cdots & 2{\mathbb{N}}P\left(2,{\mathbb{N}}\right)\end{array}\end{array}& \begin{array}{cc}\begin{array}{c}\ddots \\ \dots \end{array}& \begin{array}{c}\vdots \\ \begin{array}{ccc}{\mathbb{N}}^{2}P\left({\mathbb{N}},1\right)& \cdots & {\mathbb{N}}^{2}P\left({\mathbb{N}},{\mathbb{N}}\right)\\ \vdots & \ddots & \vdots \\ {\mathbb{N}}^{2}P\left({\mathbb{N}},1\right)& \cdots & {\mathbb{N}}^{2}P\left({\mathbb{N}},{\mathbb{N}}\right)\end{array}\end{array}\end{array}\end{array}\right)}_{{\mathbb{N}}^{2}\times {\mathbb{N}}^{2}},$$

$${\mathcal{F}}_{21}={\left(\begin{array}{ccc}0& 0& \begin{array}{cc}\dots & 0\end{array}\\ 0& 0& \begin{array}{cc}\dots & 0\end{array}\\ \begin{array}{c}\vdots \\ 0\end{array}& \begin{array}{c}\vdots \\ 0\end{array}& \begin{array}{cc}\begin{array}{c}\ddots \\ \dots \end{array}& \begin{array}{c}\vdots \\ 0\end{array}\end{array}\end{array}\right)}_{{\mathbb{N}}^{2}\times {\mathbb{N}}^{2}},$$

$${\mathcal{F}}_{22}=\frac{{\beta }_{2}}{\langle l\rangle }{\left(\begin{array}{ccc}\begin{array}{ccc}P\left(\mathrm{1,1}\right)& \cdots & P\left(1,{\mathbb{N}}\right)\\ \vdots & \ddots & \vdots \\ P\left(\mathrm{1,1}\right)& \cdots & P\left(1,{\mathbb{N}}\right)\end{array}& \begin{array}{ccc}2P\left(\mathrm{2,1}\right)& \cdots & 2P\left(2,{\mathbb{N}}\right)\\ \vdots & \ddots & \vdots \\ 2P\left(\mathrm{2,1}\right)& \cdots & 2P\left(2,{\mathbb{N}}\right)\end{array}& \begin{array}{cc}\dots & \begin{array}{ccc}{\mathbb{N}}P\left({\mathbb{N}},1\right)& \cdots & {\mathbb{N}}P\left({\mathbb{N}},{\mathbb{N}}\right)\\ \vdots & \ddots & \vdots \\ {\mathbb{N}}P\left({\mathbb{N}},1\right)& \cdots & {\mathbb{N}}P\left({\mathbb{N}},{\mathbb{N}}\right)\end{array}\end{array}\\ \begin{array}{ccc}2P\left(\mathrm{1,1}\right)& \cdots & 2P\left(1,{\mathbb{N}}\right)\\ \vdots & \ddots & \vdots \\ 2P\left(\mathrm{1,1}\right)& \cdots & 2P\left(1,{\mathbb{N}}\right)\end{array}& \begin{array}{ccc}{2}^{2}P\left(\mathrm{2,1}\right)& \cdots & {2}^{2}P\left(2,{\mathbb{N}}\right)\\ \vdots & \ddots & \vdots \\ {2}^{2}P\left(\mathrm{2,1}\right)& \cdots & {2}^{2}P\left(2,{\mathbb{N}}\right)\end{array}& \begin{array}{cc}\dots & \begin{array}{ccc}2{\mathbb{N}}P\left({\mathbb{N}},1\right)& \cdots & 2{\mathbb{N}}P\left({\mathbb{N}},{\mathbb{N}}\right)\\ \vdots & \ddots & \vdots \\ 2{\mathbb{N}}P\left({\mathbb{N}},1\right)& \cdots & 2{\mathbb{N}}P\left({\mathbb{N}},{\mathbb{N}}\right)\end{array}\end{array}\\ \begin{array}{c}\vdots \\ \begin{array}{ccc}{\mathbb{N}}P\left(\mathrm{1,1}\right)& \cdots & {\mathbb{N}}P\left(1,{\mathbb{N}}\right)\\ \vdots & \ddots & \vdots \\ {\mathbb{N}}P\left(\mathrm{1,1}\right)& \cdots & {\mathbb{N}}P\left(1,{\mathbb{N}}\right)\end{array}\end{array}& \begin{array}{c}\vdots \\ \begin{array}{ccc}2{\mathbb{N}}P\left(\mathrm{2,1}\right)& \cdots & 2{\mathbb{N}}P\left(2,{\mathbb{N}}\right)\\ \vdots & \ddots & \vdots \\ 2{\mathbb{N}}P\left(\mathrm{2,1}\right)& \cdots & 2{\mathbb{N}}P\left(2,{\mathbb{N}}\right)\end{array}\end{array}& \begin{array}{cc}\begin{array}{c}\ddots \\ \dots \end{array}& \begin{array}{c}\vdots \\ \begin{array}{ccc}{\mathbb{N}}^{2}P\left({\mathbb{N}},1\right)& \cdots & {\mathbb{N}}^{2}P\left({\mathbb{N}},{\mathbb{N}}\right)\\ \vdots & \ddots & \vdots \\ {\mathbb{N}}^{2}P\left({\mathbb{N}},1\right)& \cdots & {\mathbb{N}}^{2}P\left({\mathbb{N}},{\mathbb{N}}\right)\end{array}\end{array}\end{array}\end{array}\right)}_{{\mathbb{N}}^{2}\times {\mathbb{N}}^{2}},$$

The following matrix $V$ represents the rates of transferring out of and into the two compartments ${I}_{\left(k,m\right)}^{h}$ and ${I}_{\left(l,m\right)}^{c}$:

7 $$V={\left(\begin{array}{cc}{\mathcal{V}}_{11}& {\mathcal{V}}_{12}\\ {\mathcal{V}}_{21}& {\mathcal{V}}_{22}\end{array}\right)}_{2{\mathbb{N}}^{2}\times 2{\mathbb{N}}^{2}},$$

where ${\mathcal{V}}_{11},{\mathcal{V}}_{12},{\mathcal{V}}_{21}$ and ${\mathcal{V}}_{22}$ are ${\mathbb{N}}^{2}\times {\mathbb{N}}^{2}$ matrices given by

$${\mathcal{V}}_{11}={\left(\begin{array}{ccc}{\gamma }_{1}+{b}_{1}& 0& \begin{array}{cc}\dots & 0\end{array}\\ 0& {\gamma }_{1}+{b}_{1}& \begin{array}{cc}\dots & 0\end{array}\\ \begin{array}{c}\vdots \\ 0\end{array}& \begin{array}{c}\vdots \\ 0\end{array}& \begin{array}{cc}\begin{array}{c}\ddots \\ \dots \end{array}& \begin{array}{c}\vdots \\ {\gamma }_{1}+{b}_{1}\end{array}\end{array}\end{array}\right)}_{{\mathbb{N}}^{2}\times {\mathbb{N}}^{2}}$$

$${\mathcal{V}}_{12}={\left(\begin{array}{ccc}0& 0& \begin{array}{cc}\dots & 0\end{array}\\ 0& 0& \begin{array}{cc}\dots & 0\end{array}\\ \begin{array}{c}\vdots \\ 0\end{array}& \begin{array}{c}\vdots \\ 0\end{array}& \begin{array}{cc}\begin{array}{c}\ddots \\ \dots \end{array}& \begin{array}{c}\vdots \\ 0\end{array}\end{array}\end{array}\right)}_{{\mathbb{N}}^{2}\times {\mathbb{N}}^{2}},{\mathcal{V}}_{21}={\left(\begin{array}{ccc}0& 0& \begin{array}{cc}\dots & 0\end{array}\\ 0& 0& \begin{array}{cc}\dots & 0\end{array}\\ \begin{array}{c}\vdots \\ 0\end{array}& \begin{array}{c}\vdots \\ 0\end{array}& \begin{array}{cc}\begin{array}{c}\ddots \\ \dots \end{array}& \begin{array}{c}\vdots \\ 0\end{array}\end{array}\end{array}\right)}_{{\mathbb{N}}^{2}\times {\mathbb{N}}^{2}}$$

$${\mathcal{V}}_{22}={\left(\begin{array}{ccc}{\gamma }_{2}+{b}_{2}& 0& \begin{array}{cc}\dots & 0\end{array}\\ 0& {\gamma }_{2}+{b}_{2}& \begin{array}{cc}\dots & 0\end{array}\\ \begin{array}{c}\vdots \\ 0\end{array}& \begin{array}{c}\vdots \\ 0\end{array}& \begin{array}{cc}\begin{array}{c}\ddots \\ \dots \end{array}& \begin{array}{c}\vdots \\ {\gamma }_{2}+{b}_{2}\end{array}\end{array}\end{array}\right)}_{{\mathbb{N}}^{2}\times {\mathbb{N}}^{2}}.$$

The basic reproductive number is given by the dominant eigenvalue of $F{V}^{-1}$, where $F$ is the matrix in (6) and ${V}^{-1}$ is the inverse of the matrix in (7) calculated at the disease-free equilibrium point ${E}_{0}$.

Setting $U=F{V}^{-1}$, where $$U={\left(\begin{array}{cc}{\mathcal{U}}_{11}& {\mathcal{U}}_{12}\\ {\mathcal{U}}_{21}& {\mathcal{U}}_{22}\end{array}\right)}_{2{\mathbb{N}}^{2}\times 2{\mathbb{N}}^{2}}$$, the elements ${\mathcal{U}}_{ij}$ can be expressed by

$${\mathcal{U}}_{11}=\frac{{\beta }_{1}}{\langle k\rangle ({\gamma }_{1}+{b}_{1})}{\left(\begin{array}{ccc}\begin{array}{ccc}P\left(\mathrm{1,1}\right)& \cdots & P\left(1,{\mathbb{N}}\right)\\ \vdots & \ddots & \vdots \\ P\left(\mathrm{1,1}\right)& \cdots & P\left(1,{\mathbb{N}}\right)\end{array}& \begin{array}{ccc}2P\left(\mathrm{2,1}\right)& \cdots & 2P\left(2,{\mathbb{N}}\right)\\ \vdots & \ddots & \vdots \\ 2P\left(\mathrm{2,1}\right)& \cdots & 2P\left(2,{\mathbb{N}}\right)\end{array}& \begin{array}{cc}\dots & \begin{array}{ccc}{\mathbb{N}}P\left({\mathbb{N}},1\right)& \cdots & {\mathbb{N}}P\left({\mathbb{N}},{\mathbb{N}}\right)\\ \vdots & \ddots & \vdots \\ {\mathbb{N}}P\left({\mathbb{N}},1\right)& \cdots & {\mathbb{N}}P\left({\mathbb{N}},{\mathbb{N}}\right)\end{array}\end{array}\\ \begin{array}{ccc}2P\left(\mathrm{1,1}\right)& \cdots & 2P\left(1,{\mathbb{N}}\right)\\ \vdots & \ddots & \vdots \\ 2P\left(\mathrm{1,1}\right)& \cdots & 2P\left(1,{\mathbb{N}}\right)\end{array}& \begin{array}{ccc}{2}^{2}P\left(\mathrm{2,1}\right)& \cdots & {2}^{2}P\left(2,{\mathbb{N}}\right)\\ \vdots & \ddots & \vdots \\ {2}^{2}P\left(\mathrm{2,1}\right)& \cdots & {2}^{2}P\left(2,{\mathbb{N}}\right)\end{array}& \begin{array}{cc}\dots & \begin{array}{ccc}2{\mathbb{N}}P\left({\mathbb{N}},1\right)& \cdots & 2{\mathbb{N}}P\left({\mathbb{N}},{\mathbb{N}}\right)\\ \vdots & \ddots & \vdots \\ 2{\mathbb{N}}P\left({\mathbb{N}},1\right)& \cdots & 2{\mathbb{N}}P\left({\mathbb{N}},{\mathbb{N}}\right)\end{array}\end{array}\\ \begin{array}{c}\vdots \\ \begin{array}{ccc}{\mathbb{N}}P\left(\mathrm{1,1}\right)& \cdots & {\mathbb{N}}P\left(1,{\mathbb{N}}\right)\\ \vdots & \ddots & \vdots \\ {\mathbb{N}}P\left(1,1\right)& \cdots & {\mathbb{N}}P\left(1,{\mathbb{N}}\right)\end{array}\end{array}& \begin{array}{c}\vdots \\ \begin{array}{ccc}2{\mathbb{N}}P\left(\mathrm{2,1}\right)& \cdots & 2{\mathbb{N}}P\left(2,{\mathbb{N}}\right)\\ \vdots & \ddots & \vdots \\ 2{\mathbb{N}}P\left(\mathrm{2,1}\right)& \cdots & 2{\mathbb{N}}P\left(2,{\mathbb{N}}\right)\end{array}\end{array}& \begin{array}{cc}\begin{array}{c}\ddots \\ \dots \end{array}& \begin{array}{c}\vdots \\ \begin{array}{ccc}{\mathbb{N}}^{2}P\left({\mathbb{N}},1\right)& \cdots & {\mathbb{N}}^{2}P\left({\mathbb{N}},{\mathbb{N}}\right)\\ \vdots & \ddots & \vdots \\ {\mathbb{N}}^{2}P\left({\mathbb{N}},1\right)& \cdots & {\mathbb{N}}^{2}P\left({\mathbb{N}},{\mathbb{N}}\right)\end{array}\end{array}\end{array}\end{array}\right)}_{{\mathbb{N}}^{2}\times {\mathbb{N}}^{2}}$$

$${\mathcal{U}}_{12}=\frac{{\beta }_{3}}{\langle m\rangle ({\gamma }_{2}+{b}_{2})}{\left(\begin{array}{ccc}\begin{array}{ccc}P\left(\mathrm{1,1}\right)& \cdots & P\left(1,{\mathbb{N}}\right)\\ \vdots & \ddots & \vdots \\ P\left(\mathrm{1,1}\right)& \cdots & P\left(1,{\mathbb{N}}\right)\end{array}& \begin{array}{ccc}2P\left(\mathrm{2,1}\right)& \cdots & 2P\left(2,{\mathbb{N}}\right)\\ \vdots & \ddots & \vdots \\ 2P\left(\mathrm{2,1}\right)& \cdots & 2P\left(2,{\mathbb{N}}\right)\end{array}& \begin{array}{cc}\dots & \begin{array}{ccc}{\mathbb{N}}P\left({\mathbb{N}},1\right)& \cdots & {\mathbb{N}}P\left({\mathbb{N}},{\mathbb{N}}\right)\\ \vdots & \ddots & \vdots \\ {\mathbb{N}}P\left({\mathbb{N}},1\right)& \cdots & {\mathbb{N}}P\left({\mathbb{N}},{\mathbb{N}}\right)\end{array}\end{array}\\ \begin{array}{ccc}2P\left(\mathrm{1,1}\right)& \cdots & 2P\left(1,{\mathbb{N}}\right)\\ \vdots & \ddots & \vdots \\ 2P\left(\mathrm{1,1}\right)& \cdots & 2P\left(1,{\mathbb{N}}\right)\end{array}& \begin{array}{ccc}{2}^{2}P\left(\mathrm{2,1}\right)& \cdots & {2}^{2}P\left(2,{\mathbb{N}}\right)\\ \vdots & \ddots & \vdots \\ {2}^{2}P\left(\mathrm{2,1}\right)& \cdots & {2}^{2}P\left(2,{\mathbb{N}}\right)\end{array}& \begin{array}{cc}\dots & \begin{array}{ccc}2{\mathbb{N}}P\left({\mathbb{N}},1\right)& \cdots & 2{\mathbb{N}}P\left({\mathbb{N}},{\mathbb{N}}\right)\\ \vdots & \ddots & \vdots \\ 2{\mathbb{N}}P\left({\mathbb{N}},1\right)& \cdots & 2{\mathbb{N}}P\left({\mathbb{N}},{\mathbb{N}}\right)\end{array}\end{array}\\ \begin{array}{c}\vdots \\ \begin{array}{ccc}{\mathbb{N}}P\left(\mathrm{1,1}\right)& \cdots & {\mathbb{N}}P\left(1,{\mathbb{N}}\right)\\ \vdots & \ddots & \vdots \\ {\mathbb{N}}P\left(\mathrm{1,1}\right)& \cdots & {\mathbb{N}}P\left(1,{\mathbb{N}}\right)\end{array}\end{array}& \begin{array}{c}\vdots \\ \begin{array}{ccc}2{\mathbb{N}}P\left(\mathrm{2,1}\right)& \cdots & 2{\mathbb{N}}P\left(2,{\mathbb{N}}\right)\\ \vdots & \ddots & \vdots \\ 2{\mathbb{N}}P\left(\mathrm{2,1}\right)& \cdots & 2{\mathbb{N}}P\left(2,{\mathbb{N}}\right)\end{array}\end{array}& \begin{array}{cc}\begin{array}{c}\ddots \\ \dots \end{array}& \begin{array}{c}\vdots \\ \begin{array}{ccc}{\mathbb{N}}^{2}P\left({\mathbb{N}},1\right)& \cdots & {\mathbb{N}}^{2}P\left({\mathbb{N}},{\mathbb{N}}\right)\\ \vdots & \ddots & \vdots \\ {\mathbb{N}}^{2}P\left({\mathbb{N}},1\right)& \cdots & {\mathbb{N}}^{2}P\left({\mathbb{N}},{\mathbb{N}}\right)\end{array}\end{array}\end{array}\end{array}\right)}_{{\mathbb{N}}^{2}\times {\mathbb{N}}^{2}},$$

$${\mathcal{U}}_{21}={\left(\begin{array}{ccc}0& 0& \begin{array}{cc}\dots & 0\end{array}\\ 0& 0& \begin{array}{cc}\dots & 0\end{array}\\ \begin{array}{c}\vdots \\ 0\end{array}& \begin{array}{c}\vdots \\ 0\end{array}& \begin{array}{cc}\begin{array}{c}\ddots \\ \dots \end{array}& \begin{array}{c}\vdots \\ 0\end{array}\end{array}\end{array}\right)}_{{\mathbb{N}}^{2}\times {\mathbb{N}}^{2}},$$

$${\mathcal{U}}_{22}=\frac{{\beta }_{2}}{\langle l\rangle ({\gamma }_{2}+{b}_{2})}{\left(\begin{array}{ccc}\begin{array}{ccc}P\left(\mathrm{1,1}\right)& \cdots & P\left(1,{\mathbb{N}}\right)\\ \vdots & \ddots & \vdots \\ P\left(\mathrm{1,1}\right)& \cdots & P\left(1,{\mathbb{N}}\right)\end{array}& \begin{array}{ccc}2P\left(\mathrm{2,1}\right)& \cdots & 2P\left(2,{\mathbb{N}}\right)\\ \vdots & \ddots & \vdots \\ 2P\left(\mathrm{2,1}\right)& \cdots & 2P\left(2,{\mathbb{N}}\right)\end{array}& \begin{array}{cc}\dots & \begin{array}{ccc}{\mathbb{N}}P\left({\mathbb{N}},1\right)& \cdots & {\mathbb{N}}P\left({\mathbb{N}},{\mathbb{N}}\right)\\ \vdots & \ddots & \vdots \\ {\mathbb{N}}P\left({\mathbb{N}},1\right)& \cdots & {\mathbb{N}}P\left({\mathbb{N}},{\mathbb{N}}\right)\end{array}\end{array}\\ \begin{array}{ccc}2P\left(\mathrm{1,1}\right)& \cdots & 2P\left(1,{\mathbb{N}}\right)\\ \vdots & \ddots & \vdots \\ 2P\left(\mathrm{1,1}\right)& \cdots & 2P\left(1,{\mathbb{N}}\right)\end{array}& \begin{array}{ccc}{2}^{2}P\left(\mathrm{2,1}\right)& \cdots & {2}^{2}P\left(2,{\mathbb{N}}\right)\\ \vdots & \ddots & \vdots \\ {2}^{2}P\left(\mathrm{2,1}\right)& \cdots & {2}^{2}P\left(2,{\mathbb{N}}\right)\end{array}& \begin{array}{cc}\dots & \begin{array}{ccc}2{\mathbb{N}}P\left({\mathbb{N}},1\right)& \cdots & 2{\mathbb{N}}P\left({\mathbb{N}},{\mathbb{N}}\right)\\ \vdots & \ddots & \vdots \\ 2{\mathbb{N}}P\left({\mathbb{N}},1\right)& \cdots & 2{\mathbb{N}}P\left({\mathbb{N}},{\mathbb{N}}\right)\end{array}\end{array}\\ \begin{array}{c}\vdots \\ \begin{array}{ccc}{\mathbb{N}}P\left(\mathrm{1,1}\right)& \cdots & {\mathbb{N}}P\left(1,{\mathbb{N}}\right)\\ \vdots & \ddots & \vdots \\ {\mathbb{N}}P\left(\mathrm{1,1}\right)& \cdots & {\mathbb{N}}P\left(1,{\mathbb{N}}\right)\end{array}\end{array}& \begin{array}{c}\vdots \\ \begin{array}{ccc}2{\mathbb{N}}P\left(\mathrm{2,1}\right)& \cdots & 2{\mathbb{N}}P\left(2,{\mathbb{N}}\right)\\ \vdots & \ddots & \vdots \\ 2{\mathbb{N}}P\left(\mathrm{2,1}\right)& \cdots & 2{\mathbb{N}}P\left(2,{\mathbb{N}}\right)\end{array}\end{array}& \begin{array}{cc}\begin{array}{c}\ddots \\ \dots \end{array}& \begin{array}{c}\vdots \\ \begin{array}{ccc}{\mathbb{N}}^{2}P\left({\mathbb{N}},1\right)& \cdots & {\mathbb{N}}^{2}P\left({\mathbb{N}},{\mathbb{N}}\right)\\ \vdots & \ddots & \vdots \\ {\mathbb{N}}^{2}P\left({\mathbb{N}},1\right)& \cdots & {\mathbb{N}}^{2}P\left({\mathbb{N}},{\mathbb{N}}\right)\end{array}\end{array}\end{array}\end{array}\right)}_{{\mathbb{N}}^{2}\times {\mathbb{N}}^{2}}.$$

The characteristic equation for the $2{\mathbb{N}}^{2}$ eigenvalues $\lambda$ of matrix $U$ is

$${\left(\lambda -\frac{\langle {k}^{2}\rangle }{\langle k\rangle }\frac{{\beta }_{1}}{\left({\gamma }_{1}+{b}_{1}\right)}\right)}^{{\mathbb{N}}^{2}}{\left(\lambda -\frac{\langle {l}^{2}\rangle }{\langle l\rangle }\frac{{\beta }_{2}}{({\gamma }_{2}+{b}_{2})}\right)}^{{\mathbb{N}}^{2}}=0.$$

Therefore, we have ${\mathbb{N}}^{2}$ equal eigenvalues ${\lambda }_{1{\mathbb{N}}^{2}}=\frac{\langle {k}^{2}\rangle }{\langle k\rangle }\frac{{\beta }_{1}}{\left({\gamma }_{1}+{b}_{1}\right)}$ and another ${\mathbb{N}}^{2}$ equal eigenvalues ${\lambda }_{2{\mathbb{N}}^{2}}=\frac{\langle {l}^{2}\rangle }{\langle l\rangle }\frac{{\beta }_{2}}{({\gamma }_{2}+{b}_{2})}$, which are equivalent to the threshold values defined in (3) and (4). We cannot determine which value of the eigenvalues is larger, so we have the following Remark.

#### Remark 2

If ${\mathcal{R}}_{0}^{h}\le {\mathcal{R}}_{0}^{c}<1$ or ${\mathcal{R}}_{0}^{c}\le {\mathcal{R}}_{0}^{h}<1$, then only the disease-free equilibrium point ${E}_{0}$ exists, which confirms (i) in Theorem 1.

### Local stability analysis

The local stability analysis of the equilibrium points ${E}_{0},{E}_{1}$ and ${E}_{2}$ is proven similarly as in^25^. First, system (2) can be reduced as follows:

8 $$\begin{aligned} & {}_{0}^{C} D_{t}^{\alpha } I_{{\left( {k,m} \right)}}^{h} \left( t \right) = \beta_{1} k\left( {1 - I_{{\left( {k,m} \right)}}^{h} \left( t \right)} \right){\Theta }_{1} \left( t \right) + \beta_{3} m\left( {1 - I_{{\left( {k,m} \right)}}^{h} \left( t \right)} \right)\left( t \right){\Theta }_{3} \left( t \right)e^{{ - \omega {\Theta }_{3} \left( t \right)}} - \left( {b_{1} + \gamma_{1} } \right)I_{{\left( {k,m} \right)}}^{h} \left( t \right), \\ & {}_{0}^{C} D_{t}^{\alpha } I_{{\left( {l,m} \right)}}^{c} \left( t \right) = \beta_{2} l\left( {1 - I_{{\left( {l,m} \right)}}^{c} \left( t \right)} \right){\Theta }_{2} \left( t \right) - \left( {b_{2} + \gamma_{2} } \right)I_{{\left( {l,m} \right)}}^{c} \left( t \right) \\ \end{aligned}$$

The following theorems give the local stability analysis of the equilibrium points.

#### Theorem 2

For system (8), if ${\mathcal{R}}_{0}^{c}<1$ and ${\mathcal{R}}_{0}^{h}<1$, then the disease-free equilibrium point ${E}_{0}$ is locally asymptotically stable.

#### Theorem 3

For system (8), if ${\mathcal{R}}_{0}^{c}>1$ and ${\widehat{\mathcal{R}}}_{0}^{h}>1$, then the endemic situation ${E}_{1}$ is locally asymptotically stable.

#### Theorem 4

For system (8), if ${\mathcal{R}}_{0}^{c}<1$ and ${\mathcal{R}}_{0}^{h}>1$, then the human endemic situation ${E}_{2}$ is locally asymptotically stable.

Detailed proofs of Theorems 2 to 4 are described in the Supplementary material (Appendix B, C and D).

## Numerical simulation

The Adams predictor–corrector method is used in this section to solve system (2). Our focus will be on the curve of infected humans. The probability distribution is selected as $P\left(i,j\right)={\nu }_{o}{i}^{{\nu }_{1}}{j}^{{\nu }_{2}}$, where the constant ${\nu }_{o}$ satisfies

$$\sum_{j=1}^{\mathbb{N}}\sum_{i=1}^{\mathbb{N}}P\left(i,j\right)=1$$

and $2<{\nu }_{1},{\nu }_{2}<3$. Taking ${\nu }_{1}={\nu }_{2}=2.3$ and ${\mathbb{N}}=20$, the following examples illustrate Theorems 2 to 4.

### Example 1

**Figure 2:**
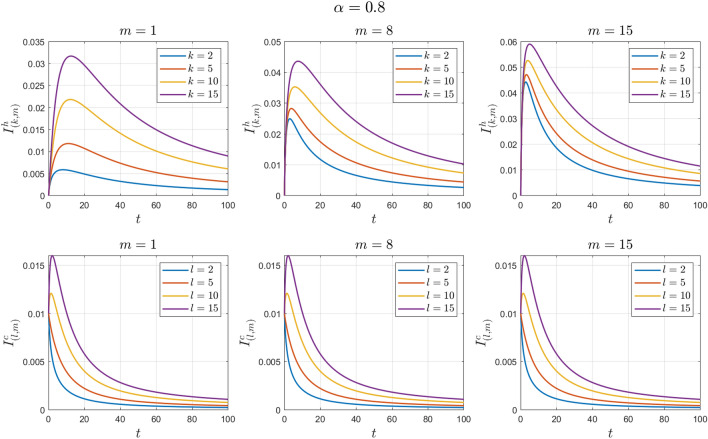
Numerical solutions of Example 1 for $\alpha =0.8$.

**Figure 3:**
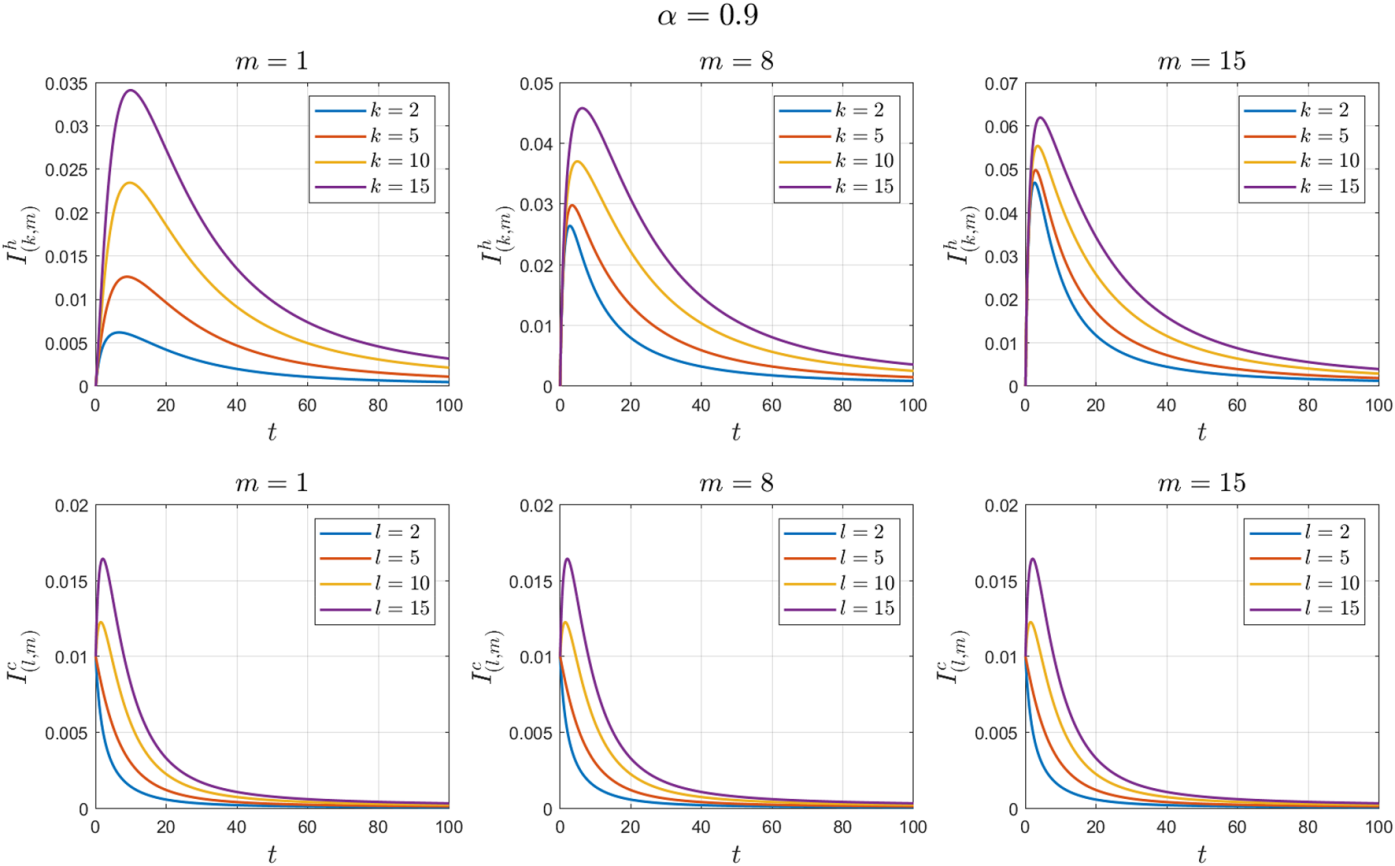
Numerical solutions of Example 1 for $\alpha =0.9$.

The values of the system parameters are chosen as ${\beta }_{1}=0.1 , {\beta }_{2}=0.1 ,{\beta }_{3}=0.4 ,{b}_{1}=0.1 ,{b}_{2}=0.2 ,{\gamma }_{1}=0.4 ,{\gamma }_{2}=0.4$ and $\omega =0.1$. Suppose the initial conditions are equal to ${S}^{h}\left(0\right)=1 , {I}^{h}\left(0\right)=0 , {S}^{c}\left(0\right)=0.99 , {I}^{c}\left(0\right)=0.01 \forall k,l,m$. We obtain ${\mathcal{R}}_{0}^{h}=0.8456$ and ${\mathcal{R}}_{0}^{c}=0.7467$. With these values, according to Theorem 2, the disease-free equilibrium point ${E}_{0}$ is locally asymptotically stable. See Figs. 2 and 3.

### Example 2

**Figure 4:**
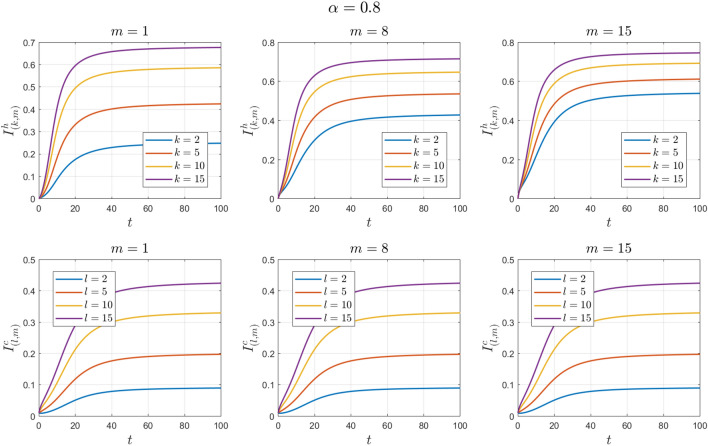
Numerical solutions of Example 2 for $\alpha =0.8$.

**Figure 5:**
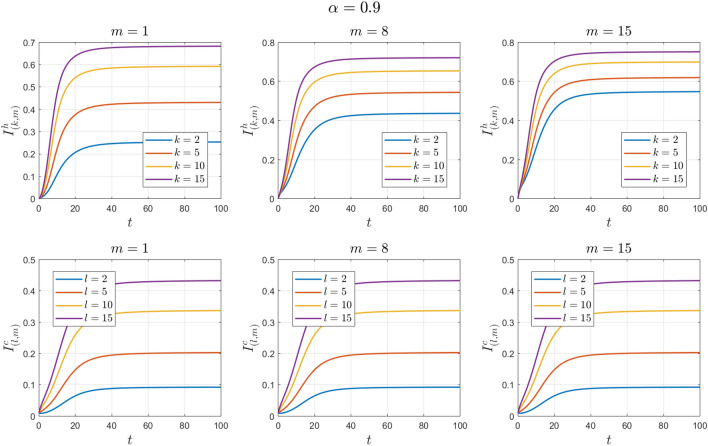
Numerical solutions of Example 2 for $\alpha =0.9$.

The values of the system parameters are chosen as ${\beta }_{1}=0.2 , {\beta }_{2}=0.2 ,{\beta }_{3}=0.4 ,{b}_{1}=0.1 ,{b}_{2}=0.2 ,{\gamma }_{1}=0.4 ,{\gamma }_{2}=0.4$ and $\omega =0.1$. Suppose the initial condition is ${S}^{h}\left(0\right)=1 , {I}^{h}\left(0\right)=0 , {S}^{c}\left(0\right)=0.99 , {I}^{c}\left(0\right)=0.01 \forall k,l,m$. We obtain ${\mathcal{R}}_{0}^{h}=1.6912$ and ${\mathcal{R}}_{0}^{c}=1.4093$. With these values, according to Theorem 3, the endemic equilibrium point ${E}_{1}$ is locally asymptotically stable. See Figs. 4 and 5.

### Example 3

**Figure 6:**
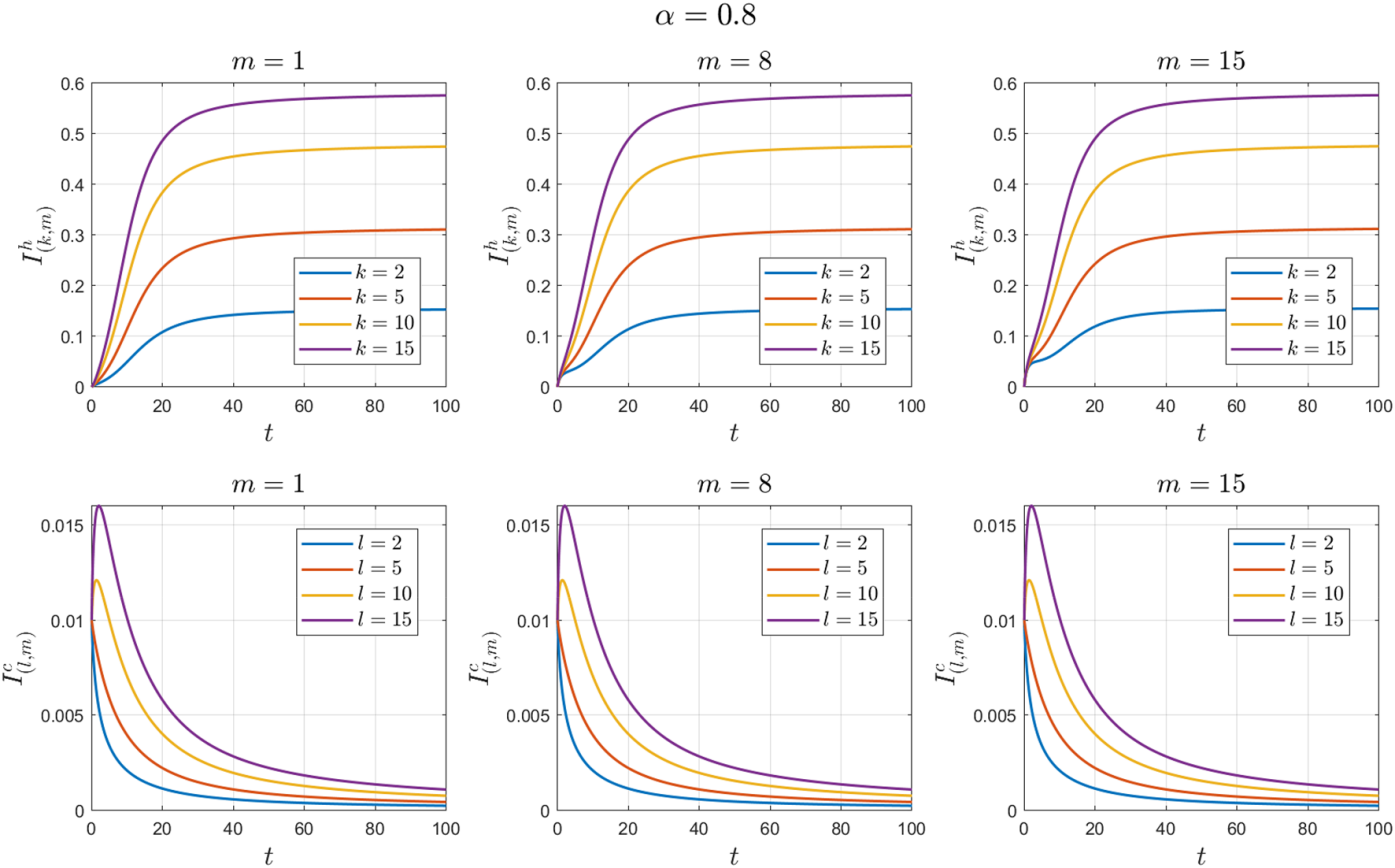
Numerical solutions of Example 3 for $\alpha =0.8$.

**Figure 7:**
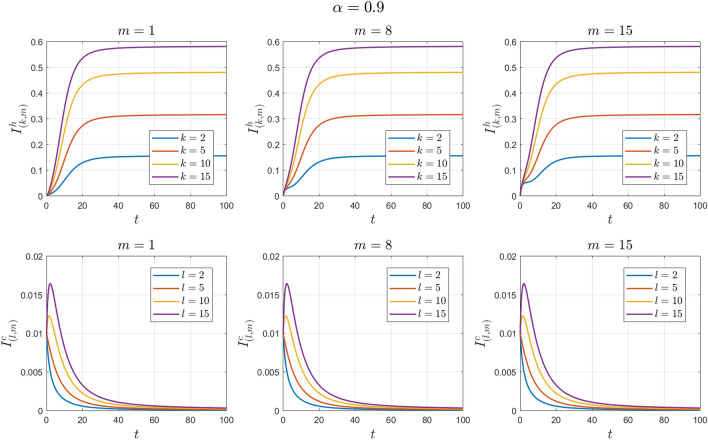
Numerical solutions of Example 3 for $\alpha =0.9$.

The values of the system parameters are chosen as ${\beta }_{1}=0.2 , {\beta }_{2}=0.1 ,{\beta }_{3}= 0.4,{b}_{1}= 0.1,{b}_{2}=0.2 ,{\gamma }_{1}=0.4 ,{\gamma }_{2}= 0.4$ and $\omega =$ 0.1. Suppose the initial condition is ${S}^{h}\left(0\right)=1 , {I}^{h}\left(0\right)=0 , {S}^{c}\left(0\right)=0.99 , {I}^{c}\left(0\right)=0.01 \forall k,l,m$. We obtain ${\mathcal{R}}_{0}^{h}=1.6912$ and ${\mathcal{R}}_{0}^{c}=0.7047$. With these values, according to Theorem 4, the human endemic equilibrium point ${E}_{2}$ is locally asymptotically stable. See Figs. 6 and 7.

### Example 4

We choose the values of the parameters as in Example 1 and change only the initial values of ${S}_{\left(k,m\right)}^{h}\left(t\right),{I}_{\left(k,m\right)}^{h}\left(t\right),{S}_{\left(l,m\right)}^{c}\left(t\right)$ and ${I}_{\left(l,m\right)}^{c}\left(t\right)$. Here, we consider three initial conditions:

$$\left\{{S}_{\left(k,m\right)}^{h}(0),{I}_{\left(k,m\right)}^{h}(0),{S}_{\left(l,m\right)}^{c}(0),{I}_{\left(l,m\right)}^{c}(0)\right\}=$$ $$\left\{\mathrm{0.5,0.5,0.5,0.5}\right\}, \left\{\mathrm{0,1},\mathrm{0,1}\right\}$$ and $$\left\{\mathrm{1,0},\mathrm{0,1}\right\}$$.

**Figure 8:**
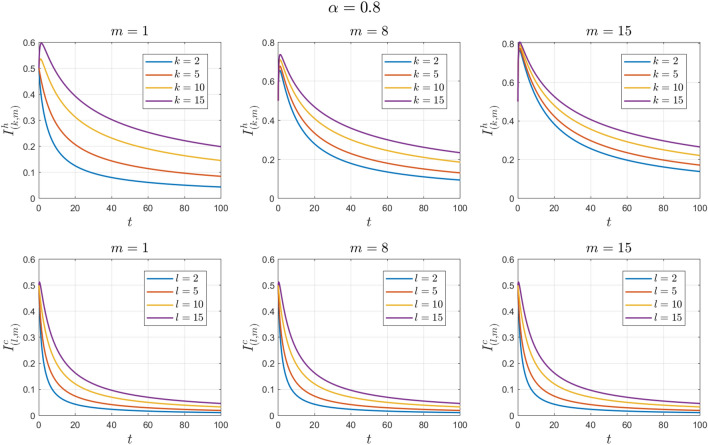
Numerical solutions of Example 4 for $\mathrm{\alpha }=0.8$ with $\left\{{S}_{\left(k,m\right)}^{h}(0),{I}_{\left(k,m\right)}^{h}(0),{S}_{\left(l,m\right)}^{c}(0),{I}_{\left(l,m\right)}^{c}(0)\right\}=\{\mathrm{0.5,0.5,0.5,0.5}\}.$

**Figure 9:**
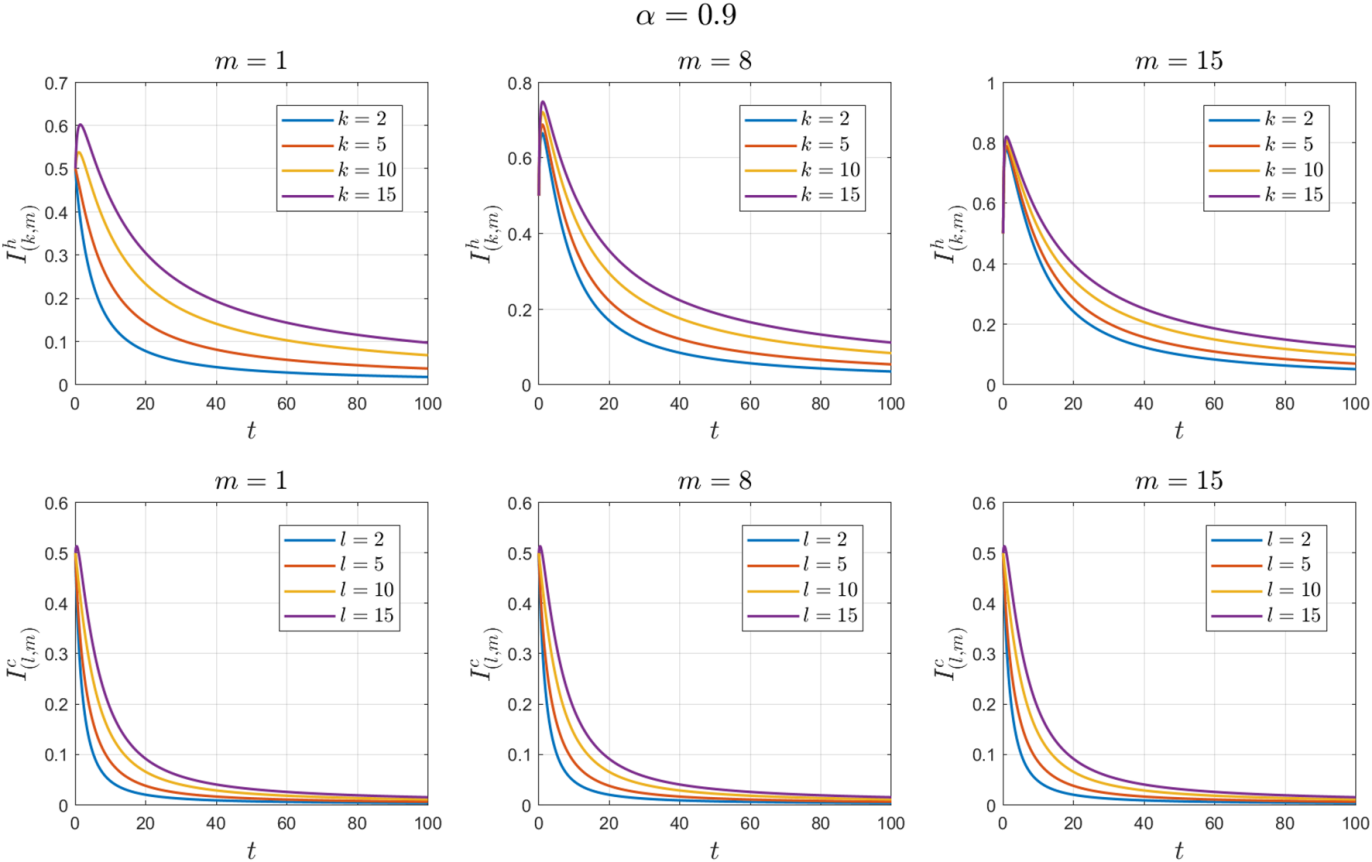
Numerical solutions of Example 4 for $\alpha =0.9$ with $\left\{{S}_{\left(k,m\right)}^{h}(0),{I}_{\left(k,m\right)}^{h}(0),{S}_{\left(l,m\right)}^{c}(0),{I}_{\left(l,m\right)}^{c}(0)\right\}=\{\mathrm{0.5,0.5,0.5,0.5}\}.$

**Figure 10:**
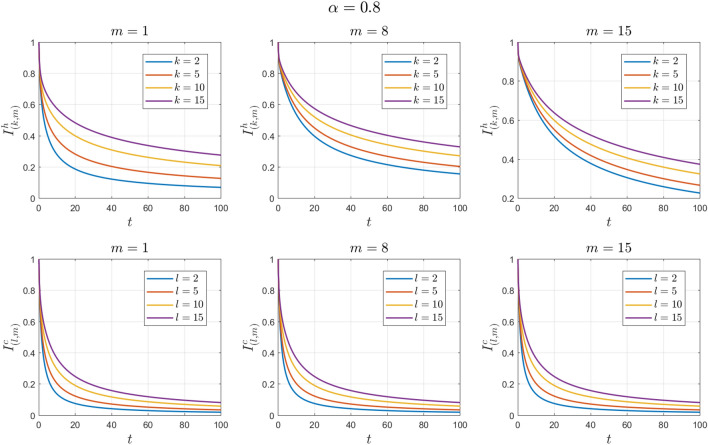
Numerical solutions of Example 4 for $\alpha =0.8$ with $\left\{{S}_{\left(k,m\right)}^{h}(0),{I}_{\left(k,m\right)}^{h}(0),{S}_{\left(l,m\right)}^{c}(0),{I}_{\left(l,m\right)}^{c}(0)\right\}=\{\mathrm{0,1},\mathrm{0,1}\}.$

**Figure 11:**
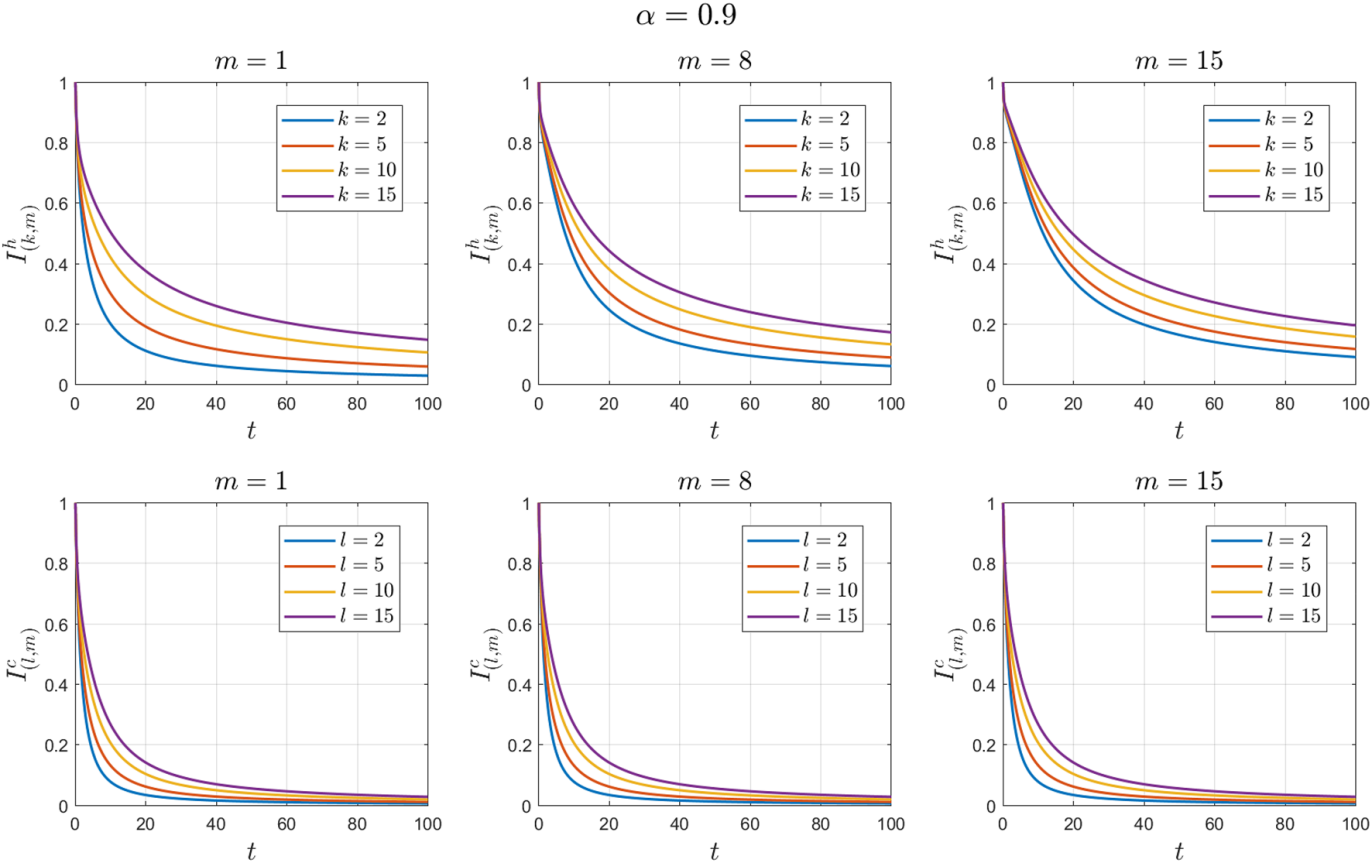
Numerical solutions of Example 4 for $\alpha =0.9$ with $\left\{{S}_{\left(k,m\right)}^{h}(0),{I}_{\left(k,m\right)}^{h}(0),{S}_{\left(l,m\right)}^{c}(0),{I}_{\left(l,m\right)}^{c}(0)\right\}=\{\mathrm{0,1},\mathrm{0,1}\}.$

**Figure 12:**
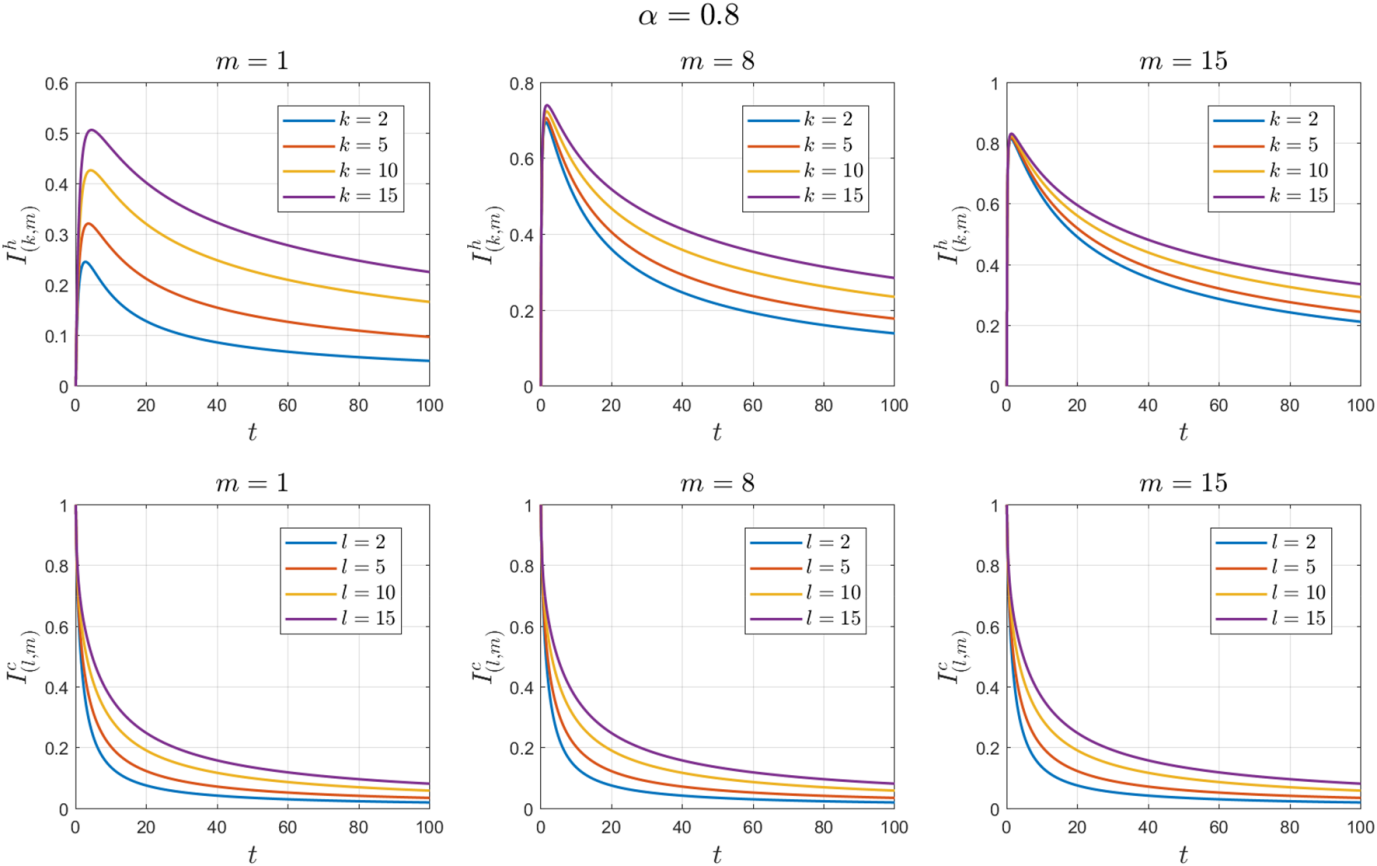
Numerical solutions of Example 4 for $\alpha =0.8$ with $\left\{{S}_{\left(k,m\right)}^{h}(0),{I}_{\left(k,m\right)}^{h}(0),{S}_{\left(l,m\right)}^{c}(0),{I}_{\left(l,m\right)}^{c}(0)\right\}=\{\mathrm{1,0},\mathrm{0,1}\}.$

**Figure 13:**
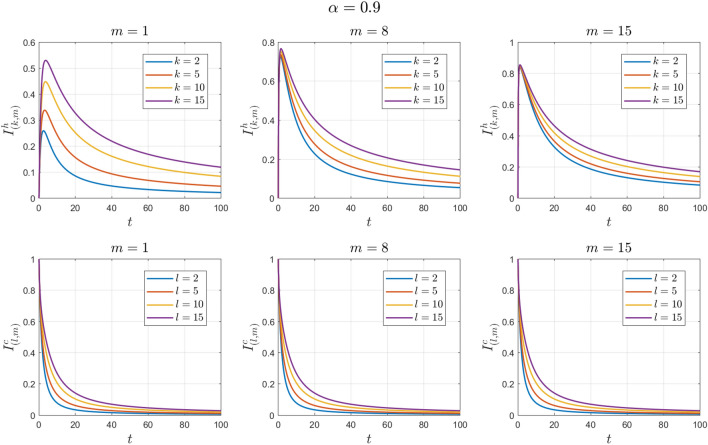
Numerical solutions of Example 4 for $\alpha =0.9$ with $\left\{{S}_{\left(k,m\right)}^{h}(0),{I}_{\left(k,m\right)}^{h}(0),{S}_{\left(l,m\right)}^{c}(0),{I}_{\left(l,m\right)}^{c}(0)\right\}=\{\mathrm{1,0},\mathrm{0,1}\}.$

With these initial conditions, the disease-free equilibrium point ${E}_{0}$ is locally asymptotically stable. See Figs. 8, 9, 10, 11, 12 and 13.

### Example 5

**Figure 14:**
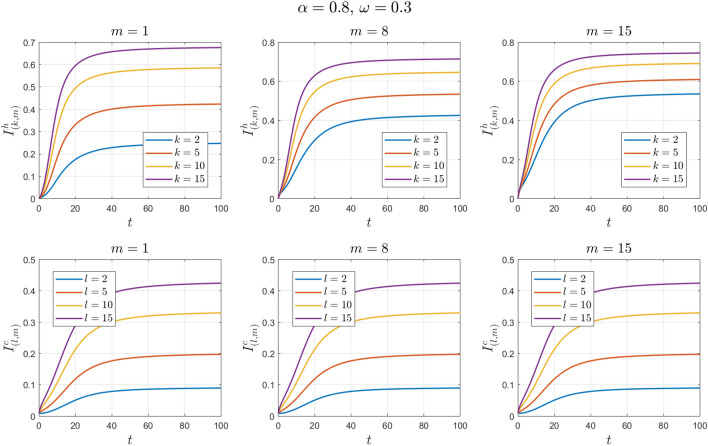
Numerical solutions of Example 5 for $\alpha =0.8$ and $\omega =0.3$.

**Figure 15:**
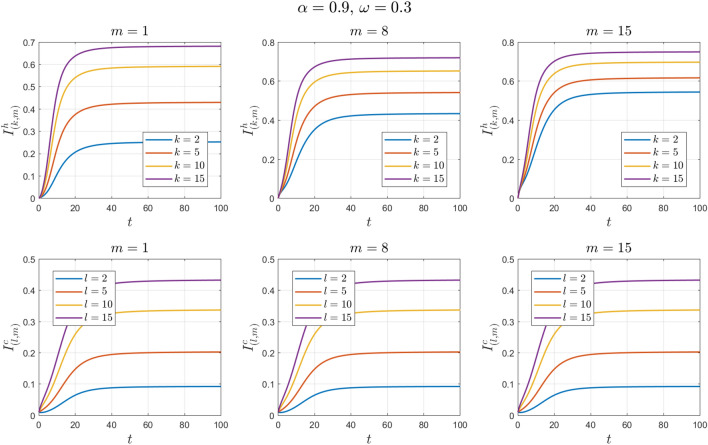
Numerical solutions of Example 5 for $\alpha =0.9$ and $\omega =0.3$.

**Figure 16:**
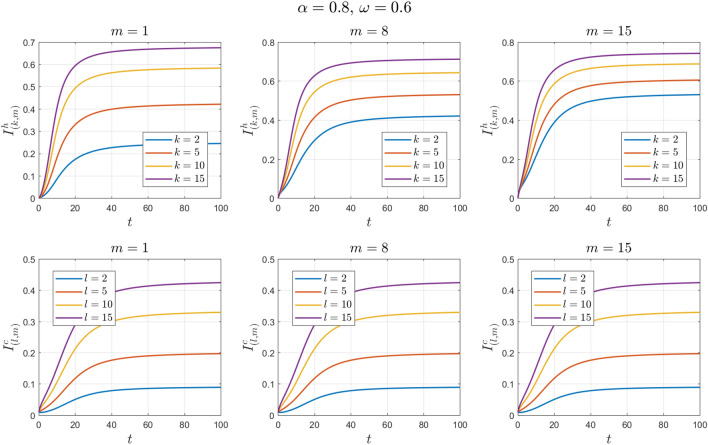
Numerical solutions of Example 5 for $\alpha =0.8$ and $\omega =0.6$.

**Figure 17:**
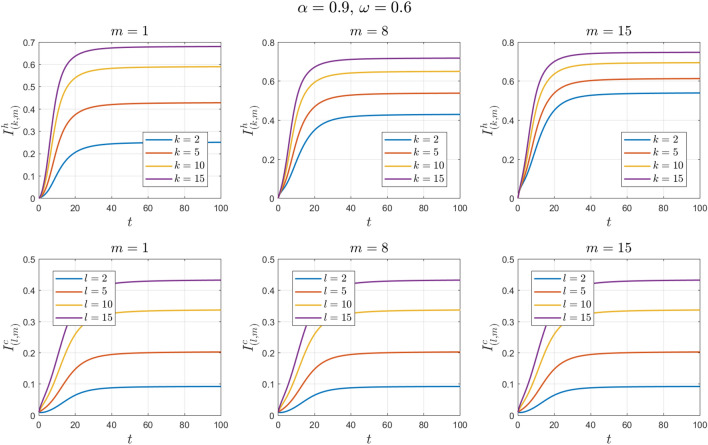
Numerical solutions of Example 5 for $\alpha =0.9$ and $\omega =0.6$.

**Figure 18:**
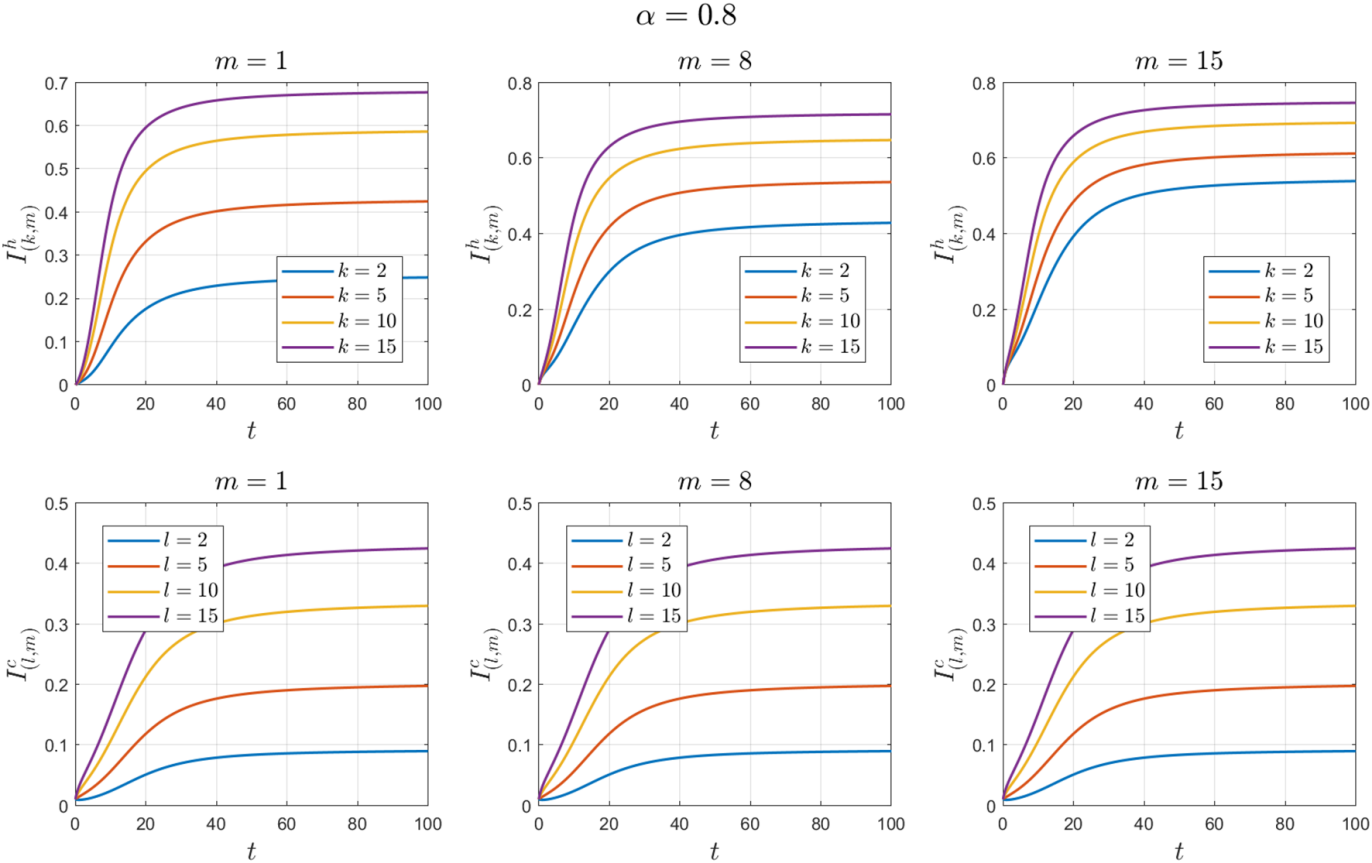
Numerical solutions of Example 5 for $\mathrm{\alpha }=0.8$ and $\upomega =0.9$.

**Figure 19:**
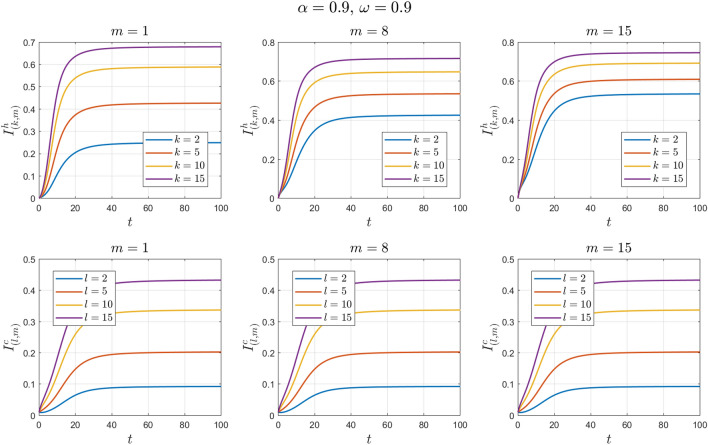
Numerical solutions of Example 5 for $\alpha =0.9$ and $\omega =0.9$.

The values of the parameters are chosen as in Example 2 except for $\omega$. This value changes to $\omega =0.3 , 0.6, 0.9$. See Figs. 14, 15, 16, 17, 18 and 19.

We can see that the fractional-order solution has a lower and wider peak than the integer-order solution. The lower the value of the fractional order is, the lower the peak of the curve. We also note the extent of the impact of adherence to preventive measures in directly dealing with camels. We find that the higher the adherence rate is, the smaller the number of infected individuals.

## Conclusion

This work presents a new mathematical formulation with heterogeneous networks employing fractional orders in differentiation for the simulation of a realistic situation during an outbreak of Middle East respiratory syndrome. This model shows the extent to which the virus outbreak is associated with the epidemiological parameters of the animal source causing the infection.

The mathematical validity of the model is verified by showing the conditions at three equilibrium positions. We calculated the threshold for the spread of the virus using the next-generation method, which resulted in two values for the threshold. The first value, ${\mathcal{R}}_{0}^{c}$, represents the threshold for spreading the virus in the camel population, and the second value, ${\mathcal{R}}_{0}^{h}$, represents the threshold for spreading the virus in the human population. We found that the type of stability for each epidemiological situation depended on the values of both ${\mathcal{R}}_{0}^{c}$ and ${\mathcal{R}}_{0}^{h}$. Finally, we used the predictor–corrector method to carry out the numerical simulation, illustrating it with many examples.

Furthermore, it became clear from several numerical experiments that the extent of the effect changes with the value of the fractional order of the model and the value of the degree in each network. This work shows that the impact on the spread of the virus can be reduced by adhering to measures to prevent infection while dealing with the animal source.

## Supplementary Information

Supplementary Information.

## Data Availability

All data generated or analyzed during this study are included in this published article. The datasets used and/or analysed during the current study available from the corresponding author on reasonable request.
