# Supplementary material for: Fractional model for Middle East respiratory syndrome coronavirus on a complex heterogeneous network
Source: Sci Rep. 2022 Dec 1;12:20706. doi: 10.1038/s41598-022-24814-1 (PMC9713123; doi:10.1038/s41598-022-24814-1)
Supplement: Supplementary file 1 — Supplementary Information. [file 41598_2022_24814_MOESM1_ESM.docx]

**Appendices**

**Appendix A.**

**Proof of Theorem 1.**

To calculate the equilibrium points for Model (2), we set all the fractional derivatives of Model (2) to zero:

$$\left\{ \begin{aligned} {}_{0}^{C}{D_{t}^{\alpha}}S_{\left( k,m \right)}^{h}\left( t \right)=0, \\ {}_{0}^{C}{D_{t}^{\alpha}}I_{\left( k,m \right)}^{h}\left( t \right)=0, \\ \begin{matrix} {}_{0}^{C}{D_{t}^{\alpha}}S_{\left( l,m \right)}^{c}\left( t \right)=0, \\ {}_{0}^{C}{D_{t}^{\alpha}}I_{\left( l,m \right)}^{c}\left( t \right)=0, \end{matrix} \end{aligned} \right.$$

After solving the last four equations, considering $\Theta_{1}\left( t \right), \Theta_{2}\left( t \right)$ and $\Theta_{3}\left( t \right)$ as additional parameters, we obtain three equilibrium points:

(i) The disease-free equilibrium points

$$E_{0}=\left\{ 1,0,1,0 \right\}_{\begin{aligned} \forall\left( k,m \right) \in\Omega_{1} \\ \forall\left( l,m \right) \in\Omega_{2} \end{aligned}}$$

(ii) The endemic equilibrium point $E_{1}=\left\{ S_{\left( k,m \right)}^{h^{*}},I_{\left( k,m \right)}^{h^{*}},S_{\left( l,m \right)}^{c^{*}},I_{\left( l,m \right)}^{c^{*}} \right\}_{\begin{aligned} \forall\left( k,m \right) \in\Omega_{1} \\ \forall\left( l,m \right) \in\Omega_{2} \end{aligned}},$ where

$$S_{\left( k,m \right)}^{h^{*}}=\frac{\gamma_{1}+b_{1}}{\beta_{1}k\Theta_{1}^{*}+\beta_{3}m\Theta_{3}^{*}e^{-\omega\Theta_{3}^{*}}+\gamma_{1}+b_{1}},$$

$$I_{\left( k,m \right)}^{h^{*}}=\frac{\beta_{1}k\Theta_{1}^{*}+\beta_{3}m\Theta_{3}^{*}e^{-\omega\Theta_{3}^{*}}}{\beta_{1}k\Theta_{1}^{*}+\beta_{3}m\Theta_{3}^{*}e^{-\omega\Theta_{3}^{*}}+\gamma_{1}+b_{1}},$$

$$S_{\left( l,m \right)}^{c^{*}}=\frac{\gamma_{2}+b_{2}}{\beta_{2}l\Theta_{2}^{*}+\gamma_{2}+b_{2}}, (A1)$$

$$I_{\left( l,m \right)}^{c^{*}}=\frac{\beta_{2}l\Theta_{2}^{*}}{\beta_{2}l\Theta_{2}^{*}+\gamma_{2}+b_{2}}.$$

(iii) The human endemic equilibrium point

$$E_{2}=\left\{ S_{\left( k,m \right)}^{h^{**}},I_{\left( k,m \right)}^{h^{**}},1,0 \right\}_{\begin{aligned} \forall\left( k,m \right) \in\Omega_{1} \\ \forall\left( l,m \right) \in\Omega_{2} \end{aligned}},$$

where

$$S_{\left( k,m \right)}^{h^{**}}=\frac{\gamma_{1}+b_{1}}{\beta_{1}k\Theta_{1}^{*}+\gamma_{1}+b_{1}},$$

$$I_{\left( k,m \right)}^{h^{**}}=\frac{\beta_{1}k\Theta_{1}^{*}}{\beta_{1}k\Theta_{1}^{*}+\gamma_{1}+b_{1}}.$$

Now, we need to determine when the values of $\Theta_{1}^{*},\Theta_{2}^{*}$ and $\Theta_{3}^{*}$ exist. Since

$\Theta_{2}\left( t \right)=\frac{1}{\left\langle\left. l \right\rangle\right.}\sum_{m=1}^{\mathbb{N}} \sum_{l=1}^{\mathbb{N}} lP(l,m) I_{\left( l,m \right)}^{c}(t)$, substituting $I_{\left( l,m \right)}^{c^{*}}$ in (3) into $\Theta_{2}\left( t \right)$, we have

$$\Theta_{2}\left( t \right)=\frac{1}{\left\langle\left. l \right\rangle\right.}\sum_{m=1}^{\mathbb{N}} \sum_{l=1}^{\mathbb{N}} lP\left( l,m \right)\frac{\beta_{2}l\Theta_{2}\left( t \right)}{\beta_{2}l\Theta_{2}\left( t \right)+\gamma_{2}+b_{2}}=g\left( \Theta_{2} \right). (A2)$$

To solve the self-consistency equation (A2), we need to prove that $g\left( \Theta_{2} \right)$ is a monotonically increasing function. Since $g\left( \Theta_{2} \right)=0$ at $\Theta_{2}=0$ and $\lim_{\Theta_{2}\to\infty} g\left( \Theta_{2} \right)=1$, the first two derivatives of $g\left( \Theta_{2} \right)$ with respect to $\Theta_{2}$ are equal to

$$\frac{dg}{d\Theta_{2}}=\frac{1}{\left\langle\left. l \right\rangle\right.}\sum_{m=1}^{\mathbb{N}} \sum_{l=1}^{\mathbb{N}} l^{2}P\left( l,m \right)\frac{\beta_{2}(\gamma_{2}+b_{2})}{{(\beta_{2}l\Theta_{2}+\gamma_{2}+b_{2})}^{2}}>0$$

and

$$\frac{d^{2}g}{d\Theta_{2}^{2}}=\frac{(-2)}{\left\langle\left. l \right\rangle\right.}\sum_{m=1}^{\mathbb{N}} \sum_{l=1}^{\mathbb{N}} l^{3}P\left( l,m \right)\frac{{\beta_{2}}^{2}(\gamma_{2}+b_{2})}{{(\beta_{2}l\Theta_{2}+\gamma_{2}+b_{2})}^{3}}<0.$$

Then, the function $g\left( \Theta_{2} \right)$ is monotonically increasing. A nonzero value for $\Theta_{2}^{*}$ exists if and only if $\left. \frac{dg}{d\Theta_{2}} \right|_{\Theta_{2}=0}>1$, i.e.,

$$\frac{\left\langle\left. l^{2} \right\rangle\right.}{\left\langle\left. l \right\rangle\right.}\frac{\beta_{2}}{(\gamma_{2}+b_{2})}>1.$$

Then,

$$\mathcal{R}_{0}^{c}=\frac{\left\langle\left. l^{2} \right\rangle\right.}{\left\langle\left. l \right\rangle\right.}\frac{\beta_{2}}{(\gamma_{2}+b_{2})}>1 (A3)$$

as a threshold value.

Similarly, for $\Theta_{3}^{*}$, we have

$$\Theta_{3}\left( t \right)=\frac{1}{\left\langle\left. m \right\rangle\right.}\sum_{m=1}^{\mathbb{N}} \sum_{l=1}^{\mathbb{N}} mP(l,m) I_{\left( l,m \right)}^{c}(t).$$

Substituting $I_{\left( l,m \right)}^{c^{*}}$ in (A1) into $\Theta_{3}\left( t \right)$, we obtain

$$\Theta_{3}\left( t \right)=\frac{1}{\left\langle\left. m \right\rangle\right.}\sum_{m=1}^{\mathbb{N}} \sum_{l=1}^{\mathbb{N}} mP(l,m)\frac{\beta_{2}l\Theta_{2}\left( t \right)}{\beta_{2}l\Theta_{2}\left( t \right)+\gamma_{2}+b_{2}}=Q\left( \Theta_{2}\left( t \right) \right).$$

It is clear that the value of $\Theta_{3}\left( t \right)$ basically depends on $\Theta_{2}\left( t \right)$. In other words, if the value of $\Theta_{2}$ exists, $\Theta_{3}$ is defined. Additionally, if $\Theta_{2}=0$, we obtain $\Theta_{3}=0$. The function $\Theta_{3}\left( t \right)=Q(\Theta_{2}\left( t \right))$ always has an increasing value with respect to $\Theta_{2}$ when $l,m$ increase.

Finally, for $\Theta_{1}^{*}$, we have

$$\Theta_{1}\left( t \right)=\frac{1}{\left\langle\left. k \right\rangle\right.}\sum_{m=1}^{\mathbb{N}} \sum_{k=1}^{\mathbb{N}} kP(k,m) I_{\left( k,m \right)}^{h}(t).$$

After substituting the value of $I_{\left( k,m \right)}^{h^{*}}$ into $\Theta_{1}\left( t \right)$, we obtain

$$\Theta_{1}\left( t \right)=\frac{1}{\left\langle\left. k \right\rangle\right.}\sum_{m=1}^{\mathbb{N}} \sum_{k=1}^{\mathbb{N}} kP(k,m) \frac{\beta_{1}k\Theta_{1}\left( t \right)+\beta_{3}m\Theta_{3}\left( t \right)e^{-\omega\Theta_{3}\left( t \right)}}{\beta_{1}k\Theta_{1}\left( t \right)+\beta_{3}m\Theta_{3}\left( t \right)e^{-\omega\Theta_{3}\left( t \right)}+\gamma_{1}+b_{1}}. (A4)$$

First, we let $\Theta_{1}\left( t \right)=f\left( \Theta_{1}\left( t \right),\Theta_{3}\left( t \right) \right),$ where

$$f\left( \Theta_{1}\left( t \right),\Theta_{3}\left( t \right) \right)=\frac{1}{\left\langle\left. k \right\rangle\right.}\sum_{m=1}^{\mathbb{N}} \sum_{k=1}^{\mathbb{N}} kP(k,m) \frac{\beta_{1}k\Theta_{1}\left( t \right)+\beta_{3}m\Theta_{3}\left( t \right)e^{-\omega\Theta_{3}\left( t \right)}}{\beta_{1}k\Theta_{1}\left( t \right)+\beta_{3}m\Theta_{3}\left( t \right)e^{-\omega\Theta_{3}\left( t \right)}+\gamma_{1}+b_{1}}.$$

We will show that the function $f\left( \Theta_{1},\Theta_{3} \right)$ is a monotonically increasing bounded function. The first and second partial derivatives of $f\left( \Theta_{1},\Theta_{3} \right)$ with respect to $\Theta_{1}$ are given by

$$\frac{\partial f}{\partial\Theta_{1}}=\frac{1}{\left\langle\left. k \right\rangle\right.}\sum_{m=1}^{\mathbb{N}} \sum_{k=1}^{\mathbb{N}} k^{2}P(k,m) \frac{\beta_{1}\left( \gamma_{1}+b_{1} \right)}{\left( \beta_{1}k\Theta_{1}+\beta_{3}m\Theta_{3}\left( t \right)e^{-\omega\Theta_{3}\left( t \right)}+\gamma_{1}+b_{1} \right)^{2}}>0$$

and

$$\frac{\partial^{2}f}{\partial\Theta_{1}^{2}}=\frac{(-2)}{\left\langle\left. k \right\rangle\right.}\sum_{m=1}^{\mathbb{N}} \sum_{k=1}^{\mathbb{N}} k^{3}P(k,m) \frac{{\beta_{1}}^{2}\left( \gamma_{1}+b_{1} \right)}{\left( \beta_{1}k\Theta_{1}+\beta_{3}m\Theta_{3}\left( t \right)e^{-\omega\Theta_{3}\left( t \right)}+\gamma_{1}+b_{1} \right)^{3}}<0.$$

Since $f\left( 0, 0 \right)=0$ and it is easy to prove that $\lim_{\Theta_{1}\to\infty} f\left( \Theta_{1},\Theta_{3} \right)=1$, the function $f\left( \Theta_{1},\Theta_{3} \right)$ is a monotonically increasing bounded function. A nontrivial solution exists for (A4) if and only if $\left. \frac{\partial f}{\partial\Theta_{1}} \right|_{\Theta_{1}=0}>1$, i.e.,

$$\frac{1}{\left\langle\left. k \right\rangle\right.}\sum_{m=1}^{\mathbb{N}} \sum_{k=1}^{\mathbb{N}} k^{2}P(k,m) \frac{\beta_{1}\left( \gamma_{1}+b_{1} \right)}{\left( \beta_{3}m\Theta_{3}\left( t \right)e^{-\omega\Theta_{3}\left( t \right)}+\gamma_{1}+b_{1} \right)^{2}}>1.$$

Then,

$${\hat{\mathcal{R}}}_{0}^{h}=\frac{1}{\left\langle\left. k \right\rangle\right.}\sum_{m=1}^{\mathbb{N}} \sum_{k=1}^{\mathbb{N}} k^{2}P(k,m) \frac{\beta_{1}\left( \gamma_{1}+b_{1} \right)}{\left( \beta_{3}m\Theta_{3}\left( t \right)e^{-\omega\Theta_{3}\left( t \right)}+\gamma_{1}+b_{1} \right)^{2}}>1, (A5)$$

as a threshold value.

We observe that when $\mathcal{R}_{0}^{c}<1$, the value of $\Theta_{3}(t)$ is equal to zero. Therefore, the threshold ${\hat{\mathcal{R}}}_{0}^{h}$ will take another form of $\mathcal{R}_{0}^{h}$:

$$\mathcal{R}_{0}^{h}=\frac{\left\langle\left. k^{2} \right\rangle\right.}{\left\langle\left. k \right\rangle\right.}\frac{\beta_{1}}{(\gamma_{1}+b_{1})}>1. (A6)$$

**Appendix B.**

**Proof of Theorem 2.**

Starting by combining the Jacobian matrix of (8) at $E_{0}$, we obtain the following $2\mathbb{N}^{2}\times2\mathbb{N}^{2}$ matrix:

$$J\left( E_{0} \right)=\left( \begin{matrix} \mathcal{I}_{11} & \mathcal{I}_{12} \\ \mathcal{I}_{21} & \mathcal{I}_{22} \end{matrix} \right)_{2\mathbb{N}^{2}\times2\mathbb{N}^{2}} (B1)$$

where each submatrix $\mathcal{I}_{ij} , 1\leq i,j\leq2$ is an $\mathbb{N}^{2}\times\mathbb{N}^{2}$ matrix and is given by

$$\mathcal{I}_{11}=\left( \begin{matrix} \begin{matrix} \beta_{1}K_{11,1}-B_{1} & \cdots& \beta_{1}K_{11,\mathbb{N}} \\ \vdots& \ddots& \vdots\\ \beta_{1}K_{11,1} & \cdots& \beta_{1}K_{11,\mathbb{N}}-B_{1} \end{matrix} & \begin{matrix} \beta_{1}K_{12,1} & \cdots& \beta_{1}K_{12,\mathbb{N}} \\ \vdots& \ddots& \vdots\\ \beta_{1}K_{12,1} & \cdots& \beta_{1}K_{12,\mathbb{N}} \end{matrix} & \begin{matrix} \ldots& \begin{matrix} \beta_{1}K_{1\mathbb{N,}1} & \cdots& \beta_{1}K_{1\mathbb{N,N}} \\ \vdots& \ddots& \vdots\\ \beta_{1}K_{1\mathbb{N,}1} & \cdots& \beta_{1}K_{1\mathbb{N,N}} \end{matrix} \end{matrix} \\ \begin{matrix} \beta_{1}K_{21,1} & \cdots& \beta_{1}K_{21,\mathbb{N}} \\ \vdots& \ddots& \vdots\\ \beta_{1}K_{21,1} & \cdots& \beta_{1}K_{21,\mathbb{N}} \end{matrix} & \begin{matrix} \beta_{1}K_{22,1}-B_{1} & \cdots& \beta_{1}K_{22,\mathbb{N}} \\ \vdots& \ddots& \vdots\\ \beta_{1}K_{22,1} & \cdots& \beta_{1}K_{22,\mathbb{N}}-B_{1} \end{matrix} & \begin{matrix} \ldots& \begin{matrix} \beta_{1}K_{2\mathbb{N,}1} & \cdots& \beta_{1}K_{2\mathbb{N,N}} \\ \vdots& \ddots& \vdots\\ \beta_{1}K_{2\mathbb{N,}1} & \cdots& \beta_{1}K_{2\mathbb{N,N}} \end{matrix} \end{matrix} \\ \begin{matrix} \vdots\\ \begin{matrix} \beta_{1}K_{\mathbb{N}1,1} & \cdots& \beta_{1}K_{\mathbb{N}1,\mathbb{N}} \\ \vdots& \ddots& \vdots\\ \beta_{1}K_{\mathbb{N}1,1} & \cdots& \beta_{1}K_{\mathbb{N}1,\mathbb{N}} \end{matrix} \end{matrix} & \begin{matrix} \vdots\\ \begin{matrix} \beta_{1}K_{\mathbb{N}2,1} & \cdots& \beta_{1}K_{\mathbb{N}2,\mathbb{N}} \\ \vdots& \ddots& \vdots\\ \beta_{1}K_{\mathbb{N}2,1} & \cdots& \beta_{1}K_{\mathbb{N}2,\mathbb{N}} \end{matrix} \end{matrix} & \begin{matrix} \begin{matrix} \ddots\\ \ldots\end{matrix} & \begin{matrix} \vdots\\ \begin{matrix} \beta_{1}K_{\mathbb{NN},1}-B_{1} & \cdots& \beta_{1}K_{\mathbb{NN,N}} \\ \vdots& \ddots& \vdots\\ \beta_{1}K_{\mathbb{NN},1} & \cdots& \beta_{1}K_{\mathbb{NN,N}}-B_{1} \end{matrix} \end{matrix} \end{matrix} \end{matrix} \right)_{\mathbb{N}^{2}\times\mathbb{N}^{2}},$$

$$\mathcal{I}_{12}=\left( \begin{matrix} \begin{matrix} \beta_{3}M_{11,1} & \cdots& \beta_{3}M_{11,\mathbb{N}} \\ \vdots& \ddots& \vdots\\ \beta_{3}M_{11,1} & \cdots& \beta_{3}M_{11,\mathbb{N}} \end{matrix} & \begin{matrix} \beta_{3}M_{12,1} & \cdots& \beta_{3}M_{12,\mathbb{N}} \\ \vdots& \ddots& \vdots\\ \beta_{3}M_{12,1} & \cdots& \beta_{3}M_{12,\mathbb{N}} \end{matrix} & \begin{matrix} \ldots& \begin{matrix} \beta_{3}M_{1\mathbb{N,}1} & \cdots& \beta_{3}M_{1\mathbb{N,N}} \\ \vdots& \ddots& \vdots\\ \beta_{3}M_{1\mathbb{N,}1} & \cdots& \beta_{3}M_{1\mathbb{N,N}} \end{matrix} \end{matrix} \\ \begin{matrix} \beta_{3}M_{21,1} & \cdots& \beta_{3}M_{21,\mathbb{N}} \\ \vdots& \ddots& \vdots\\ \beta_{3}M_{21,1} & \cdots& \beta_{3}M_{21,\mathbb{N}} \end{matrix} & \begin{matrix} \beta_{3}M_{22,1} & \cdots& \beta_{3}M_{22,\mathbb{N}} \\ \vdots& \ddots& \vdots\\ \beta_{3}M_{22,1} & \cdots& \beta_{3}M_{22,\mathbb{N}} \end{matrix} & \begin{matrix} \ldots& \begin{matrix} \beta_{3}M_{2\mathbb{N,}1} & \cdots& \beta_{3}M_{2\mathbb{N,N}} \\ \vdots& \ddots& \vdots\\ \beta_{3}M_{2\mathbb{N,}1} & \cdots& \beta_{3}M_{2\mathbb{N,N}} \end{matrix} \end{matrix} \\ \begin{matrix} \vdots\\ \begin{matrix} \beta_{3}M_{\mathbb{N}1,1} & \cdots& \beta_{3}M_{\mathbb{N}1,\mathbb{N}} \\ \vdots& \ddots& \vdots\\ \beta_{3}M_{\mathbb{N}1,1} & \cdots& \beta_{3}M_{\mathbb{N}1,\mathbb{N}} \end{matrix} \end{matrix} & \begin{matrix} \vdots\\ \begin{matrix} \beta_{3}M_{\mathbb{N}2,1} & \cdots& \beta_{3}M_{\mathbb{N}2,\mathbb{N}} \\ \vdots& \ddots& \vdots\\ \beta_{3}M_{\mathbb{N}2,1} & \cdots& \beta_{3}M_{\mathbb{N}2,\mathbb{N}} \end{matrix} \end{matrix} & \begin{matrix} \begin{matrix} \ddots\\ \ldots\end{matrix} & \begin{matrix} \vdots\\ \begin{matrix} \beta_{3}M_{\mathbb{NN},1} & \cdots& \beta_{3}M_{\mathbb{NN,N}} \\ \vdots& \ddots& \vdots\\ \beta_{3}M_{\mathbb{NN},1} & \cdots& \beta_{3}M_{\mathbb{NN,N}} \end{matrix} \end{matrix} \end{matrix} \end{matrix} \right)_{\mathbb{N}^{2}\times\mathbb{N}^{2}},$$

$$\mathcal{I}_{21}=\left( \begin{matrix} 0 & 0 & \begin{matrix} \ldots& 0 \end{matrix} \\ 0 & 0 & \begin{matrix} \ldots& 0 \end{matrix} \\ \begin{matrix} \vdots\\ 0 \end{matrix} & \begin{matrix} \vdots\\ 0 \end{matrix} & \begin{matrix} \begin{matrix} \ddots\\ \ldots\end{matrix} & \begin{matrix} \vdots\\ 0 \end{matrix} \end{matrix} \end{matrix} \right)_{\mathbb{N}^{2}\times\mathbb{N}^{2}},$$

$$\mathcal{I}_{22}=\left( \begin{matrix} \begin{matrix} \beta_{2}L_{11,1}-B_{2} & \cdots& \beta_{2}L_{11,\mathbb{N}} \\ \vdots& \ddots& \vdots\\ \beta_{2}L_{11,1} & \cdots& \beta_{2}L_{11,\mathbb{N}}-B_{2} \end{matrix} & \begin{matrix} \beta_{2}L_{12,1} & \cdots& \beta_{2}L_{12,\mathbb{N}} \\ \vdots& \ddots& \vdots\\ \beta_{2}L_{12,1} & \cdots& \beta_{2}L_{12,\mathbb{N}} \end{matrix} & \begin{matrix} \ldots& \begin{matrix} \beta_{2}L_{1\mathbb{N,}1} & \cdots& \beta_{2}L_{1\mathbb{N,N}} \\ \vdots& \ddots& \vdots\\ \beta_{2}L_{1\mathbb{N,}1} & \cdots& \beta_{2}L_{1\mathbb{N,N}} \end{matrix} \end{matrix} \\ \begin{matrix} \beta_{2}L_{21,1} & \cdots& \beta_{2}L_{21,\mathbb{N}} \\ \vdots& \ddots& \vdots\\ \beta_{2}L_{21,1} & \cdots& \beta_{2}L_{21,\mathbb{N}} \end{matrix} & \begin{matrix} \beta_{2}L_{22,1}-B_{2} & \cdots& \beta_{2}L_{22,\mathbb{N}} \\ \vdots& \ddots& \vdots\\ \beta_{2}L_{22,1} & \cdots& \beta_{2}L_{22,\mathbb{N}}-B_{2} \end{matrix} & \begin{matrix} \ldots& \begin{matrix} \beta_{2}L_{2\mathbb{N,}1} & \cdots& \beta_{2}L_{2\mathbb{N,N}} \\ \vdots& \ddots& \vdots\\ \beta_{2}L_{2\mathbb{N,}1} & \cdots& \beta_{2}L_{2\mathbb{N,N}} \end{matrix} \end{matrix} \\ \begin{matrix} \vdots\\ \begin{matrix} \beta_{2}L_{\mathbb{N}1,1} & \cdots& \beta_{2}L_{\mathbb{N}1,\mathbb{N}} \\ \vdots& \ddots& \vdots\\ \beta_{2}L_{\mathbb{N}1,1} & \cdots& \beta_{2}L_{\mathbb{N}1,\mathbb{N}} \end{matrix} \end{matrix} & \begin{matrix} \vdots\\ \begin{matrix} \beta_{2}L_{\mathbb{N}2,1} & \cdots& \beta_{2}L_{\mathbb{N}2,\mathbb{N}} \\ \vdots& \ddots& \vdots\\ \beta_{2}L_{\mathbb{N}2,1} & \cdots& \beta_{2}L_{\mathbb{N}2,\mathbb{N}} \end{matrix} \end{matrix} & \begin{matrix} \begin{matrix} \ddots\\ \ldots\end{matrix} & \begin{matrix} \vdots\\ \begin{matrix} \beta_{2}L_{\mathbb{NN},1}-B_{2} & \cdots& \beta_{2}L_{\mathbb{NN,N}} \\ \vdots& \ddots& \vdots\\ \beta_{2}L_{\mathbb{NN},1} & \cdots& \beta_{2}L_{\mathbb{NN,N}}-B_{2} \end{matrix} \end{matrix} \end{matrix} \end{matrix} \right)_{\mathbb{N}^{2}\times\mathbb{N}^{2}},$$

where

$$B_{1}=\gamma_{1}+b_{1},B_{2}=\gamma_{2}+b_{2}, K_{ij,\tau}=\frac{ijP\left( j,\tau\right)}{\left\langle\left. k \right\rangle\right.} , M_{ij,\tau}=\frac{ijP\left( j,\tau\right)}{\left\langle\left. m \right\rangle\right.} and L_{ij,\tau}=\frac{ijP\left( j,\tau\right)}{\left\langle\left. l \right\rangle\right.}\forall1\leq i,j,\tau\mathbb{\leq N.}$$

All eigenvalues of the Jacobian matrix (B1) should satisfy the following condition [19-20]:

$\left| arg(x_{i}) \right|>\frac{\alpha\pi}{2}. (B2)$

After expanding the Jacobian matrix at $E_{0}$, we obtain the following characteristic equation:

$$\left( x+B_{1} \right)^{\mathbb{N}^{2}-1}\left( x+B_{2} \right)^{\mathbb{N}^{2}-1}\left( x+B_{1}(1-\mathcal{R}_{0}^{h}) \right)\left( x+B_{2}(1-\mathcal{R}_{0}^{c}) \right)=0.$$

Clearly, we obtain $\mathbb{N}^{2}-1$ eigenvalues equal to $-(\gamma_{1}+b_{1})$ and another $\mathbb{N}^{2}-1$ eigenvalues equal to $-(\gamma_{2}+b_{2})$. We obtain two negative eigenvalues from the third and fourth brackets $\left( x+B_{1}(1-\mathcal{R}_{0}^{h}) \right)\left( x+B_{2}(1-\mathcal{R}_{0}^{c}) \right)$ if and only if $\mathcal{R}_{0}^{c}<1$ and $\mathcal{R}_{0}^{h}<1$. Therefore, all the eigenvalues of the Jacobian matrix $J\left( E_{0} \right)$ have a negative sign, which means that it satisfies condition (B2). Hence, Theorem 2 is proved.

**Appendix C**

**Proof of Theorem 3.**

To prove Theorem 3, we substitute the endemic equilibrium point $E_{1}$ into the Jacobian matrix, which takes the form

$$J\left( E_{1} \right)=\left( \begin{matrix} \mathcal{I}_{11} & \mathcal{I}_{12} \\ \mathcal{I}_{21} & \mathcal{I}_{22} \end{matrix} \right)_{2\mathbb{N}^{2}\times2\mathbb{N}^{2}}, (C1)$$

where each submatrix $\mathcal{I}_{ij} , 1\leq i,j\leq2$ is an $\mathbb{N}^{2}\times\mathbb{N}^{2}$ matrix and is given by

$$\mathcal{I}_{11}=$$

$$\left( \begin{matrix} \begin{matrix} \beta_{1}K_{11,1}\epsilon_{1}-z_{1}-g_{1}-B_{1} & \cdots& \beta_{1}K_{11,\mathbb{N}}\epsilon_{1} \\ \vdots& \ddots& \vdots\\ \beta_{1}K_{11,1}\epsilon_{1} & \cdots& \beta_{1}K_{11,\mathbb{N}}\epsilon_{1}-z_{1}-g_{\mathbb{N}}-B_{1} \end{matrix} & \begin{matrix} \beta_{1}K_{12,1}\epsilon_{1} & \cdots& \beta_{1}K_{12,\mathbb{N}}\epsilon_{1} \\ \vdots& \ddots& \vdots\\ \beta_{1}K_{12,1}\epsilon_{1} & \cdots& \beta_{1}K_{12,\mathbb{N}}\epsilon_{1} \end{matrix} & \begin{matrix} \ldots& \begin{matrix} \beta_{1}K_{1\mathbb{N,}1}\epsilon_{1} & \cdots& \beta_{1}K_{1\mathbb{N,N}}\epsilon_{1} \\ \vdots& \ddots& \vdots\\ \beta_{1}K_{1\mathbb{N,}1}\epsilon_{1} & \cdots& \beta_{1}K_{1\mathbb{N,N}}\epsilon_{1} \end{matrix} \end{matrix} \\ \begin{matrix} \beta_{1}K_{21,1}\epsilon_{2} & \cdots& \beta_{1}K_{21,\mathbb{N}}\epsilon_{2} \\ \vdots& \ddots& \vdots\\ \beta_{1}K_{21,1}\epsilon_{2} & \cdots& \beta_{1}K_{21,\mathbb{N}}\epsilon_{2} \end{matrix} & \begin{matrix} \beta_{1}K_{22,1}\epsilon_{2}-z_{2}-g_{1}-B_{1} & \cdots& \beta_{1}K_{22,\mathbb{N}}\epsilon_{2} \\ \vdots& \ddots& \vdots\\ \beta_{1}K_{22,1}\epsilon_{2} & \cdots& \beta_{1}K_{22,\mathbb{N}}\epsilon_{2}-z_{2}-g_{\mathbb{N}}-B_{1} \end{matrix} & \begin{matrix} \ldots& \begin{matrix} \beta_{1}K_{2\mathbb{N,}1}\epsilon_{2} & \cdots& \beta_{1}K_{2\mathbb{N,N}}\epsilon_{2} \\ \vdots& \ddots& \vdots\\ \beta_{1}K_{2\mathbb{N,}1}\epsilon_{2} & \cdots& \beta_{1}K_{2\mathbb{N,N}}\epsilon_{2} \end{matrix} \end{matrix} \\ \begin{matrix} \vdots\\ \begin{matrix} \beta_{1}K_{\mathbb{N}1,1}\epsilon_{\mathbb{N}} & \cdots& \beta_{1}K_{\mathbb{N}1,\mathbb{N}}\epsilon_{\mathbb{N}} \\ \vdots& \ddots& \vdots\\ \beta_{1}K_{\mathbb{N}1,1}\epsilon_{\mathbb{N}} & \cdots& \beta_{1}K_{\mathbb{N}1,\mathbb{N}}\epsilon_{\mathbb{N}} \end{matrix} \end{matrix} & \begin{matrix} \vdots\\ \begin{matrix} \beta_{1}K_{\mathbb{N}2,1}\epsilon_{\mathbb{N}} & \cdots& \beta_{1}K_{\mathbb{N}2,\mathbb{N}}\epsilon_{\mathbb{N}} \\ \vdots& \ddots& \vdots\\ \beta_{1}K_{\mathbb{N}2,1}\epsilon_{\mathbb{N}} & \cdots& \beta_{1}K_{\mathbb{N}2,\mathbb{N}}\epsilon_{\mathbb{N}} \end{matrix} \end{matrix} & \begin{matrix} \begin{matrix} \ddots\\ \ldots\end{matrix} & \begin{matrix} \vdots\\ \begin{matrix} \beta_{1}K_{\mathbb{NN},1}\epsilon_{\mathbb{N}}-z_{\mathbb{N}}-g_{1}-B_{1} & \cdots& \beta_{1}K_{\mathbb{NN,N}}\epsilon_{\mathbb{N}} \\ \vdots& \ddots& \vdots\\ \beta_{1}K_{\mathbb{NN},1}\epsilon_{\mathbb{N}} & \cdots& \beta_{1}K_{\mathbb{NN,N}}\epsilon_{\mathbb{N}}-z_{\mathbb{N}}-g_{\mathbb{N}}-B_{1} \end{matrix} \end{matrix} \end{matrix} \end{matrix} \right)_{\mathbb{N}^{2}\times\mathbb{N}^{2}},$$

$$\mathcal{I}_{12}=\left( \begin{matrix} \begin{matrix} \beta_{3}M_{11,1}\pi\epsilon_{1} & \cdots& \beta_{3}M_{11,\mathbb{N}}\pi\epsilon_{1} \\ \vdots& \ddots& \vdots\\ \beta_{3}M_{11,1}\pi\epsilon_{1} & \cdots& \beta_{3}M_{11,\mathbb{N}}\pi\epsilon_{1} \end{matrix} & \begin{matrix} \beta_{3}M_{12,1}\pi\epsilon_{1} & \cdots& \beta_{3}M_{12,\mathbb{N}}\pi\epsilon_{1} \\ \vdots& \ddots& \vdots\\ \beta_{3}M_{12,1}\pi\epsilon_{1} & \cdots& \beta_{3}M_{12,\mathbb{N}}\pi\epsilon_{1} \end{matrix} & \begin{matrix} \ldots& \begin{matrix} \beta_{3}M_{1\mathbb{N,}1}\pi\epsilon_{1} & \cdots& \beta_{3}M_{1\mathbb{N,N}}\pi\epsilon_{1} \\ \vdots& \ddots& \vdots\\ \beta_{3}M_{1\mathbb{N,}1}\pi\epsilon_{1} & \cdots& \beta_{3}M_{1\mathbb{N,N}}\pi\epsilon_{1} \end{matrix} \end{matrix} \\ \begin{matrix} \beta_{3}M_{21,1}\pi\epsilon_{2} & \cdots& \beta_{3}M_{21,\mathbb{N}}\pi\epsilon_{2} \\ \vdots& \ddots& \vdots\\ \beta_{3}M_{21,1}\pi\epsilon_{2} & \cdots& \beta_{3}M_{21,\mathbb{N}}\pi\epsilon_{2} \end{matrix} & \begin{matrix} \beta_{3}M_{22,1}\pi\epsilon_{2} & \cdots& \beta_{3}M_{22,\mathbb{N}}\pi\epsilon_{2} \\ \vdots& \ddots& \vdots\\ \beta_{3}M_{22,1}\pi\epsilon_{2} & \cdots& \beta_{3}M_{22,\mathbb{N}}\pi\epsilon_{2} \end{matrix} & \begin{matrix} \ldots& \begin{matrix} \beta_{3}M_{2\mathbb{N,}1}\pi\epsilon_{2} & \cdots& \beta_{3}M_{2\mathbb{N,N}}\pi\epsilon_{2} \\ \vdots& \ddots& \vdots\\ \beta_{3}M_{2\mathbb{N,}1}\pi\epsilon_{2} & \cdots& \beta_{3}M_{2\mathbb{N,N}}\pi\epsilon_{2} \end{matrix} \end{matrix} \\ \begin{matrix} \vdots\\ \begin{matrix} \beta_{3}M_{\mathbb{N}1,1}\pi\epsilon_{\mathbb{N}} & \cdots& \beta_{3}M_{\mathbb{N}1,\mathbb{N}}\pi\epsilon_{\mathbb{N}} \\ \vdots& \ddots& \vdots\\ \beta_{3}M_{\mathbb{N}1,1}\pi\epsilon_{\mathbb{N}} & \cdots& \beta_{3}M_{\mathbb{N}1,\mathbb{N}}\pi\epsilon_{\mathbb{N}} \end{matrix} \end{matrix} & \begin{matrix} \vdots\\ \begin{matrix} \beta_{3}M_{\mathbb{N}2,1}\pi\epsilon_{\mathbb{N}} & \cdots& \beta_{3}M_{\mathbb{N}2,\mathbb{N}}\pi\epsilon_{\mathbb{N}} \\ \vdots& \ddots& \vdots\\ \beta_{3}M_{\mathbb{N}2,1}\pi\epsilon_{\mathbb{N}} & \cdots& \beta_{3}M_{\mathbb{N}2,\mathbb{N}}\pi\epsilon_{\mathbb{N}} \end{matrix} \end{matrix} & \begin{matrix} \begin{matrix} \ddots\\ \ldots\end{matrix} & \begin{matrix} \vdots\\ \begin{matrix} \beta_{3}M_{\mathbb{NN},1}\pi\epsilon_{\mathbb{N}} & \cdots& \beta_{3}M_{\mathbb{NN,N}}\pi\epsilon_{\mathbb{N}} \\ \vdots& \ddots& \vdots\\ \beta_{3}M_{\mathbb{NN},1}\pi\epsilon_{\mathbb{N}} & \cdots& \beta_{3}M_{\mathbb{NN,N}}\pi\epsilon_{\mathbb{N}} \end{matrix} \end{matrix} \end{matrix} \end{matrix} \right)_{\mathbb{N}^{2}\times\mathbb{N}^{2}},$$

$$\mathcal{I}_{21}=\left( \begin{matrix} 0 & 0 & \begin{matrix} \ldots& 0 \end{matrix} \\ 0 & 0 & \begin{matrix} \ldots& 0 \end{matrix} \\ \begin{matrix} \vdots\\ 0 \end{matrix} & \begin{matrix} \vdots\\ 0 \end{matrix} & \begin{matrix} \begin{matrix} \ddots\\ \ldots\end{matrix} & \begin{matrix} \vdots\\ 0 \end{matrix} \end{matrix} \end{matrix} \right)_{\mathbb{N}^{2}\times\mathbb{N}^{2}},$$

$$\mathcal{I}_{22}=$$

$$\left( \begin{matrix} \begin{matrix} \beta_{2}L_{11,1}\sigma_{1}-w_{1}-B_{2} & \cdots& \beta_{2}L_{11,\mathbb{N}}\sigma_{1} \\ \vdots& \ddots& \vdots\\ \beta_{2}L_{11,1}\sigma_{1} & \cdots& \beta_{2}L_{11,\mathbb{N}}\sigma_{1}-w_{1}-B_{2} \end{matrix} & \begin{matrix} \beta_{2}L_{12,1}\sigma_{1} & \cdots& \beta_{2}L_{12,\mathbb{N}}\sigma_{1} \\ \vdots& \ddots& \vdots\\ \beta_{2}L_{12,1}\sigma_{1} & \cdots& \beta_{2}L_{12,\mathbb{N}}\sigma_{1} \end{matrix} & \begin{matrix} \ldots& \begin{matrix} \beta_{2}L_{1\mathbb{N,}1}\sigma_{1} & \cdots& \beta_{2}L_{1\mathbb{N,N}}\sigma_{1} \\ \vdots& \ddots& \vdots\\ \beta_{2}L_{1\mathbb{N,}1}\sigma_{1} & \cdots& \beta_{2}L_{1\mathbb{N,N}}\sigma_{1} \end{matrix} \end{matrix} \\ \begin{matrix} \beta_{2}L_{21,1}\sigma_{2} & \cdots& \beta_{2}L_{21,\mathbb{N}}\sigma_{2} \\ \vdots& \ddots& \vdots\\ \beta_{2}L_{21,1}\sigma_{2} & \cdots& \beta_{2}L_{21,\mathbb{N}}\sigma_{2} \end{matrix} & \begin{matrix} \beta_{2}L_{22,1}\sigma_{2}-w_{2}-B_{2} & \cdots& \beta_{2}L_{22,\mathbb{N}}\sigma_{2} \\ \vdots& \ddots& \vdots\\ \beta_{2}L_{22,1}\sigma_{2} & \cdots& \beta_{2}L_{22,\mathbb{N}}\sigma_{2}-w_{2}-B_{2} \end{matrix} & \begin{matrix} \ldots& \begin{matrix} \beta_{2}L_{2\mathbb{N,}1}\sigma_{2} & \cdots& \beta_{2}L_{2\mathbb{N,N}}\sigma_{2} \\ \vdots& \ddots& \vdots\\ \beta_{2}L_{2\mathbb{N,}1}\sigma_{2} & \cdots& \beta_{2}L_{2\mathbb{N,N}}\sigma_{2} \end{matrix} \end{matrix} \\ \begin{matrix} \vdots\\ \begin{matrix} \beta_{2}L_{\mathbb{N}1,1}\sigma_{\mathbb{N}} & \cdots& \beta_{2}L_{\mathbb{N}1,\mathbb{N}}\sigma_{\mathbb{N}} \\ \vdots& \ddots& \vdots\\ \beta_{2}L_{\mathbb{N}1,1}\sigma_{\mathbb{N}} & \cdots& \beta_{2}L_{\mathbb{N}1,\mathbb{N}}\sigma_{\mathbb{N}} \end{matrix} \end{matrix} & \begin{matrix} \vdots\\ \begin{matrix} \beta_{2}L_{\mathbb{N}2,1}\sigma_{\mathbb{N}} & \cdots& \beta_{2}L_{\mathbb{N}2,\mathbb{N}}\sigma_{\mathbb{N}} \\ \vdots& \ddots& \vdots\\ \beta_{2}L_{\mathbb{N}2,1}\sigma_{\mathbb{N}} & \cdots& \beta_{2}L_{\mathbb{N}2,\mathbb{N}}\sigma_{\mathbb{N}} \end{matrix} \end{matrix} & \begin{matrix} \begin{matrix} \ddots\\ \ldots\end{matrix} & \begin{matrix} \vdots\\ \begin{matrix} \beta_{2}L_{\mathbb{NN},1}\sigma_{\mathbb{N}}-w_{\mathbb{N}}-B_{2} & \cdots& \beta_{2}L_{\mathbb{NN,N}}\sigma_{\mathbb{N}} \\ \vdots& \ddots& \vdots\\ \beta_{2}L_{\mathbb{NN},1}\sigma_{\mathbb{N}} & \cdots& \beta_{2}L_{\mathbb{NN,N}}\sigma_{\mathbb{N}}-w_{\mathbb{N}}-B_{2} \end{matrix} \end{matrix} \end{matrix} \end{matrix} \right)_{\mathbb{N}^{2}\times\mathbb{N}^{2}},$$

where $\epsilon_{i}=S_{\left( i,m \right)}^{h} \forall m , z_{i}=\beta_{1}i\Theta_{1}^{*} , g_{\tau}=\beta_{3}\tau\Theta_{3}^{*}e^{-\omega\Theta_{3}^{*}} , \pi=e^{-\omega\Theta_{3}^{*}}\left( 1-\omega\Theta_{3}^{*} \right),$

$w_{j}=\beta_{2}j\Theta_{2}^{*} , \sigma_{j}=S_{\left( j,m \right)}^{c} \forall m.$

After expanding the Jacobian matrix at $E_{1}$, we obtain the following characteristic equation of degree ${2\mathbb{N}}^{2}$:

$$\left[ \prod_{i=1}^{\mathbb{N}} \left( x+B_{1}+z_{i}+g_{i} \right)^{\mathbb{N}}\left( 1-\sum_{i=1}^{\mathbb{N}} \frac{\beta_{1}\epsilon_{i}\sum_{m=1}^{\mathbb{N}} K_{iim}}{\left( x+B_{1}+z_{i}+g_{i} \right)} \right) \right]\left[ \prod_{i=1}^{\mathbb{N}} \left( x+B_{2}+w_{i} \right)^{\mathbb{N}}\left( 1-\sum_{i=1}^{\mathbb{N}} \frac{\beta_{2}\sigma_{i}\sum_{m=1}^{\mathbb{N}} L_{iim}}{\left( x+B_{2}+w_{i} \right)} \right) \right]=0. (C2)$$

Equation ($C2$) can be rewritten as

$$\mathcal{C}_{1}\left( x \right)\mathcal{C}_{2}\left( x \right)=0, (C3)$$

where

$$\mathcal{C}_{1}\left( x \right)=\left[ \prod_{i=1}^{\mathbb{N}} \left( x+B_{1}+z_{i}+g_{i} \right)^{\mathbb{N}}\left( 1-\sum_{i=1}^{\mathbb{N}} \frac{\beta_{1}\epsilon_{i}\sum_{m=1}^{\mathbb{N}} K_{iim}}{\left( x+B_{1}+z_{i}+g_{i} \right)} \right) \right],$$

$$\mathcal{C}_{2}\left( x \right)=\left[ \prod_{i=1}^{\mathbb{N}} \left( x+B_{2}+w_{i} \right)^{\mathbb{N}}\left( 1-\sum_{i=1}^{\mathbb{N}} \frac{\beta_{2}\sigma_{i}\sum_{m=1}^{\mathbb{N}} L_{iim}}{\left( x+B_{2}+w_{i} \right)} \right) \right].$$

From (C3), either $\mathcal{C}_{1}\left( x \right)=0$ or $\mathcal{C}_{2}\left( x \right)=0$. When $\mathcal{C}_{1}\left( x \right)=0$, we have a polynomial function of degree $\mathbb{N}^{2}$. Searching for $\mathcal{C}_{1}\left( x \right)=0$ solutions, we have the following cases. In case one,

$$\left( x+B_{1}+z_{i}+g_{i} \right)^{\mathbb{N}}=0.$$

It is clear that there are $\mathbb{N}^{2}$ eigenvalues equal to $-\left( B_{1}+z_{i}+g_{i} \right) \forall i$ that satisfy condition (B2). In the second case,

$$\prod_{i=1}^{\mathbb{N}} \left( x+B_{1}+z_{i}+g_{i} \right)^{\mathbb{N}}\left( 1-\sum_{i=1}^{\mathbb{N}} \frac{\beta_{1}\epsilon_{i}\sum_{m=1}^{\mathbb{N}} K_{iim}}{\left( x+B_{1}+z_{i}+g_{i} \right)} \right)=0,$$

which can be rewritten in the following form:

$$\left( x+\eta_{1} \right)^{\mathbb{N-}1}\left( x+\eta_{2} \right)^{\mathbb{N-}1}\ldots\left( x+\eta_{\mathbb{N}} \right)^{\mathbb{N-}1}\left[ \left( x+\eta_{1} \right)\left( x+\eta_{2} \right)\ldots\left( x+\eta_{\mathbb{N}} \right)-\beta_{1}\epsilon_{1}\sum_{m=1}^{\mathbb{N}} K_{11m}\left( x+\eta_{2} \right)\left( x+\eta_{3} \right)\ldots\left( x+\eta_{\mathbb{N}} \right)-\beta_{1}\epsilon_{2}\sum_{m=1}^{\mathbb{N}} K_{22m}\left( x+\eta_{1} \right)\left( x+\eta_{3} \right)\ldots\left( x+\eta_{\mathbb{N}} \right)-\ldots-\beta_{1}\epsilon_{\mathbb{N}}\sum_{m=1}^{\mathbb{N}} K_{\mathbb{NN}m}\left( x+\eta_{1} \right)\left( x+\eta_{2} \right)\ldots\left( x+\eta_{\mathbb{N-}1} \right) \right]=0,$$

where

$\eta_{i}=B_{1}+z_{i}+g_{i}>0$, which depends on $z_{i}$ and $g_{i}$ ($z_{i}$ and $g_{i}$ have an increasing value); i.e., $\eta_{i}<\eta_{i+1} , \forall1\leq i\mathbb{\leq N}$. From the first part,

$$\left( x+\eta_{1} \right)^{\mathbb{N-}1}\left( x+\eta_{2} \right)^{\mathbb{N-}1}\ldots\left( x+\eta_{\mathbb{N}} \right)^{\mathbb{N-}1}=0,$$

we obtain $\mathbb{N}^{2}\mathbb{-N}$ negative eigenvalues, and from the second part,

$$\mathcal{D}_{1}\left( x \right)=\left[ \left( x+\eta_{1} \right)\left( x+\eta_{2} \right)\ldots\left( x+\eta_{\mathbb{N}} \right)-\beta_{1}\epsilon_{1}\sum_{m=1}^{\mathbb{N}} K_{11m}\left( x+\eta_{2} \right)\left( x+\eta_{3} \right)\ldots\left( x+\eta_{\mathbb{N}} \right)-\beta_{1}\epsilon_{2}\sum_{m=1}^{\mathbb{N}} K_{22m}\left( x+\eta_{1} \right)\left( x+\eta_{3} \right)\ldots\left( x+\eta_{\mathbb{N}} \right)-\ldots-\beta_{1}\epsilon_{\mathbb{N}}\sum_{m=1}^{\mathbb{N}} K_{\mathbb{NN}m}\left( x+\eta_{1} \right)\left( x+\eta_{2} \right)\ldots\left( x+\eta_{\mathbb{N-}1} \right) \right]=0.$$

It is easy to check that

$$\mathcal{D}_{1}\left( -\eta_{i} \right)\mathcal{D}_{1}\left( -\eta_{i+1} \right)<0 , \forall1\leq i\mathbb{\leq N,}$$

and therefore, we have one negative root in the interval $[-\eta_{i+1},-\eta_{i}]$. We can conclude that we have $\mathbb{N-}1$ negative roots in the interval $[-\eta_{\mathbb{N}},-\eta_{1}]$. The last root can be found in the interval $[-\eta_{1},0]$, where $\mathcal{D}_{1}\left( -\eta_{1} \right)<0$ and $\mathcal{D}_{1}\left( 0 \right)>0$. Finally, we obtain that $\mathcal{D}_{1}\left( x \right)$ has $\mathbb{N}$ negative solutions in the interval $[-\eta_{\mathbb{N}},0]$, which directly satisfy condition (B2). Hence, $\mathcal{C}_{1}\left( x \right)$ has a total of $\mathbb{N}^{2}$ negative solutions.

We use the previous technique to obtain the other $\mathbb{N}^{2}$ eigenvalues from $\mathcal{C}_{2}\left( x \right)$.

We have a polynomial of degree $\mathbb{N}^{2}$ as

$$\mathcal{C}_{2}\left( x \right)=\left[ \prod_{i=1}^{\mathbb{N}} \left( x+B_{2}+w_{i} \right)^{\mathbb{N}}\left( 1-\sum_{i=1}^{\mathbb{N}} \frac{\beta_{2}\sigma_{i}\sum_{m=1}^{\mathbb{N}} L_{iim}}{\left( x+B_{2}+w_{i} \right)} \right) \right]=0.$$

In the first case, when

$\left( x+B_{2}+w_{i} \right)^{\mathbb{N}}=0$,

we obtain $\mathbb{N}^{2}$ negative eigenvalues equal to $-\left( B_{2}+w_{i} \right) \forall i$. Therefore, we have $\mathbb{N}^{2}$ negative eigenvalues that satisfy condition (B2). In the second case,

$$\left( x+\zeta_{1} \right)^{\mathbb{N-}1}\left( x+\zeta_{2} \right)^{\mathbb{N-}1}\ldots\left( x+\zeta_{\mathbb{N}} \right)^{\mathbb{N-}1}\left[ \left( x+\zeta_{1} \right)\left( x+\zeta_{2} \right)\ldots\left( x+\zeta_{\mathbb{N}} \right)-\beta_{2}\sigma_{1}\sum_{m=1}^{\mathbb{N}} L_{11m}\left( x+\zeta_{2} \right)\left( x+\zeta_{3} \right)\ldots\left( x+\zeta_{\mathbb{N}} \right)-\beta_{2}\sigma_{2}\sum_{m=1}^{\mathbb{N}} L_{22m}\left( x+\zeta_{1} \right)\left( x+\zeta_{3} \right)\ldots\left( x+\zeta_{\mathbb{N}} \right)-\ldots-\beta_{2}\sigma_{\mathbb{N}}\sum_{m=1}^{\mathbb{N}} L_{\mathbb{NN}m}\left( x+\zeta_{1} \right)\left( x+\zeta_{2} \right)\ldots\left( x+\zeta_{\mathbb{N-}1} \right) \right]=0,$$

where

$\zeta_{i}=B_{2}+w_{i}>0$, which depends on $w_{i}$ ($w_{i}$has an increasing value); i.e., $\zeta_{i}<\zeta_{i+1} , \forall1\leq i\mathbb{\leq N}$. The first part,

$$\left( x+\zeta_{1} \right)^{\mathbb{N-}1}\left( x+\zeta_{2} \right)^{\mathbb{N-}1}\ldots\left( x+\zeta_{\mathbb{N}} \right)^{\mathbb{N-}1}=0$$

yields $\mathbb{N}^{2}\mathbb{-N}$ negative eigenvalues, and from the second part,

$$\mathcal{D}_{2}\left( x \right)=\left[ \left( x+\zeta_{1} \right)\left( x+\zeta_{2} \right)\ldots\left( x+\zeta_{\mathbb{N}} \right)-\beta_{2}\sigma_{1}\sum_{m=1}^{\mathbb{N}} L_{11m}\left( x+\zeta_{2} \right)\left( x+\zeta_{3} \right)\ldots\left( x+\zeta_{\mathbb{N}} \right)-\beta_{2}\sigma_{2}\sum_{m=1}^{\mathbb{N}} L_{22m}\left( x+\zeta_{1} \right)\left( x+\zeta_{3} \right)\ldots\left( x+\zeta_{\mathbb{N}} \right)-\ldots-\beta_{2}\sigma_{\mathbb{N}}\sum_{m=1}^{\mathbb{N}} L_{\mathbb{NN}m}\left( x+\zeta_{1} \right)\left( x+\zeta_{2} \right)\ldots\left( x+\zeta_{\mathbb{N-}1} \right) \right]=0,$$

we have

$$\mathcal{D}_{2}\left( -\zeta_{i} \right)\mathcal{D}_{2}\left( -\zeta_{i+1} \right)<0 , \forall1\leq i\mathbb{\leq N.}$$

Additionally, $\mathcal{D}_{2}\left( -\zeta_{1} \right)\mathcal{D}_{2}\left( 0 \right)<0$. Finally, we obtain that $\mathcal{D}_{2}\left( x \right)$ has $\mathbb{N}$ negative solutions in the interval $[-\zeta_{\mathbb{N}},0]$. Hence, $\mathcal{C}_{2}\left( x \right)$ has $\mathbb{N}^{2}$negative solutions that directly satisfy condition (B2), so the endemic equilibrium point $E_{2}$ is locally asymptotically stable. The proof is complete.

**Appendix D**

For system (8), if $\mathcal{R}_{0}^{c}<1$ and $\mathcal{R}_{0}^{h}>1$, then the human endemic situation $E_{2}$ is locally asymptotically stable.

**Proof of Theorem 4.**

To prove Theorem 4, we substitute the human endemic equilibrium point $E_{2}$ into the Jacobian matrix, which takes the form

$$J\left( E_{2} \right)=\left( \begin{matrix} \mathcal{I}_{11} & \mathcal{I}_{12} \\ \mathcal{I}_{21} & \mathcal{I}_{22} \end{matrix} \right)_{2\mathbb{N}^{2}\times2\mathbb{N}^{2}},$$

where each submatrix $\mathcal{I}_{ij} , 1\leq i,j\leq2$ is an $\mathbb{N}^{2}\times\mathbb{N}^{2}$ matrix and is given by

$$\mathcal{I}_{11}=$$

$$\left( \begin{matrix} \begin{matrix} \beta_{1}K_{11,1}\epsilon_{1}-z_{1}-B_{1} & \cdots& \beta_{1}K_{11,\mathbb{N}}\epsilon_{1} \\ \vdots& \ddots& \vdots\\ \beta_{1}K_{11,1}\epsilon_{1} & \cdots& \beta_{1}K_{11,\mathbb{N}}\epsilon_{1}-z_{1}-B_{1} \end{matrix} & \begin{matrix} \beta_{1}K_{12,1}\epsilon_{1} & \cdots& \beta_{1}K_{12,\mathbb{N}}\epsilon_{1} \\ \vdots& \ddots& \vdots\\ \beta_{1}K_{12,1}\epsilon_{1} & \cdots& \beta_{1}K_{12,\mathbb{N}}\epsilon_{1} \end{matrix} & \begin{matrix} \ldots& \begin{matrix} \beta_{1}K_{1\mathbb{N,}1}\epsilon_{1} & \cdots& \beta_{1}K_{1\mathbb{N,N}}\epsilon_{1} \\ \vdots& \ddots& \vdots\\ \beta_{1}K_{1\mathbb{N,}1}\epsilon_{1} & \cdots& \beta_{1}K_{1\mathbb{N,N}}\epsilon_{1} \end{matrix} \end{matrix} \\ \begin{matrix} \beta_{1}K_{21,1}\epsilon_{2} & \cdots& \beta_{1}K_{21,\mathbb{N}}\epsilon_{2} \\ \vdots& \ddots& \vdots\\ \beta_{1}K_{21,1}\epsilon_{2} & \cdots& \beta_{1}K_{21,\mathbb{N}}\epsilon_{2} \end{matrix} & \begin{matrix} \beta_{1}K_{22,1}\epsilon_{2}-z_{2}-B_{1} & \cdots& \beta_{1}K_{22,\mathbb{N}}\epsilon_{2} \\ \vdots& \ddots& \vdots\\ \beta_{1}K_{22,1}\epsilon_{2} & \cdots& \beta_{1}K_{22,\mathbb{N}}\epsilon_{2}-z_{2}-B_{1} \end{matrix} & \begin{matrix} \ldots& \begin{matrix} \beta_{1}K_{2\mathbb{N,}1}\epsilon_{2} & \cdots& \beta_{1}K_{2\mathbb{N,N}}\epsilon_{2} \\ \vdots& \ddots& \vdots\\ \beta_{1}K_{2\mathbb{N,}1}\epsilon_{2} & \cdots& \beta_{1}K_{2\mathbb{N,N}}\epsilon_{2} \end{matrix} \end{matrix} \\ \begin{matrix} \vdots\\ \begin{matrix} \beta_{1}K_{\mathbb{N}1,1}\epsilon_{\mathbb{N}} & \cdots& \beta_{1}K_{\mathbb{N}1,\mathbb{N}}\epsilon_{\mathbb{N}} \\ \vdots& \ddots& \vdots\\ \beta_{1}K_{\mathbb{N}1,1}\epsilon_{\mathbb{N}} & \cdots& \beta_{1}K_{\mathbb{N}1,\mathbb{N}}\epsilon_{\mathbb{N}} \end{matrix} \end{matrix} & \begin{matrix} \vdots\\ \begin{matrix} \beta_{1}K_{\mathbb{N}2,1}\epsilon_{\mathbb{N}} & \cdots& \beta_{1}K_{\mathbb{N}2,\mathbb{N}}\epsilon_{\mathbb{N}} \\ \vdots& \ddots& \vdots\\ \beta_{1}K_{\mathbb{N}2,1}\epsilon_{\mathbb{N}} & \cdots& \beta_{1}K_{\mathbb{N}2,\mathbb{N}}\epsilon_{\mathbb{N}} \end{matrix} \end{matrix} & \begin{matrix} \begin{matrix} \ddots\\ \ldots\end{matrix} & \begin{matrix} \vdots\\ \begin{matrix} \beta_{1}K_{\mathbb{NN},1}\epsilon_{\mathbb{N}}-z_{\mathbb{N}}-B_{1} & \cdots& \beta_{1}K_{\mathbb{NN,N}}\epsilon_{\mathbb{N}} \\ \vdots& \ddots& \vdots\\ \beta_{1}K_{\mathbb{NN},1}\epsilon_{\mathbb{N}} & \cdots& \beta_{1}K_{\mathbb{NN,N}}\epsilon_{\mathbb{N}}-z_{\mathbb{N}}-B_{1} \end{matrix} \end{matrix} \end{matrix} \end{matrix} \right)_{\mathbb{N}^{2}\times\mathbb{N}^{2}}$$

$$\mathcal{I}_{12}=$$

$$\left( \begin{matrix} \begin{matrix} \beta_{3}M_{11,1}\epsilon_{1} & \cdots& \beta_{3}M_{11,\mathbb{N}}\epsilon_{1} \\ \vdots& \ddots& \vdots\\ \beta_{3}M_{11,1}\epsilon_{1} & \cdots& \beta_{3}M_{11,\mathbb{N}}\epsilon_{1} \end{matrix} & \begin{matrix} \beta_{3}M_{12,1}\epsilon_{1} & \cdots& \beta_{3}M_{12,\mathbb{N}}\epsilon_{1} \\ \vdots& \ddots& \vdots\\ \beta_{3}M_{12,1}\epsilon_{1} & \cdots& \beta_{3}M_{12,\mathbb{N}}\epsilon_{1} \end{matrix} & \begin{matrix} \ldots& \begin{matrix} \beta_{3}M_{1\mathbb{N,}1}\epsilon_{1} & \cdots& \beta_{3}M_{1\mathbb{N,N}}\epsilon_{1} \\ \vdots& \ddots& \vdots\\ \beta_{3}M_{1\mathbb{N,}1}\epsilon_{1} & \cdots& \beta_{3}M_{1\mathbb{N,N}}\epsilon_{1} \end{matrix} \end{matrix} \\ \begin{matrix} \beta_{3}M_{21,1}\epsilon_{2} & \cdots& \beta_{3}M_{21,\mathbb{N}}\epsilon_{2} \\ \vdots& \ddots& \vdots\\ \beta_{3}M_{21,1}\epsilon_{2} & \cdots& \beta_{3}M_{21,\mathbb{N}}\epsilon_{2} \end{matrix} & \begin{matrix} \beta_{3}M_{22,1}\epsilon_{2} & \cdots& \beta_{3}M_{22,\mathbb{N}}\epsilon_{2} \\ \vdots& \ddots& \vdots\\ \beta_{3}M_{22,1}\epsilon_{2} & \cdots& \beta_{3}M_{22,\mathbb{N}}\epsilon_{2} \end{matrix} & \begin{matrix} \ldots& \begin{matrix} \beta_{3}M_{2\mathbb{N,}1}\epsilon_{2} & \cdots& \beta_{3}M_{2\mathbb{N,N}}\epsilon_{2} \\ \vdots& \ddots& \vdots\\ \beta_{3}M_{2\mathbb{N,}1}\epsilon_{2} & \cdots& \beta_{3}M_{2\mathbb{N,N}}\epsilon_{2} \end{matrix} \end{matrix} \\ \begin{matrix} \vdots\\ \begin{matrix} \beta_{3}M_{\mathbb{N}1,1}\epsilon_{\mathbb{N}} & \cdots& \beta_{3}M_{\mathbb{N}1,\mathbb{N}}\epsilon_{\mathbb{N}} \\ \vdots& \ddots& \vdots\\ \beta_{3}M_{\mathbb{N}1,1}\epsilon_{\mathbb{N}} & \cdots& \beta_{3}M_{\mathbb{N}1,\mathbb{N}}\epsilon_{\mathbb{N}} \end{matrix} \end{matrix} & \begin{matrix} \vdots\\ \begin{matrix} \beta_{3}M_{\mathbb{N}2,1}\epsilon_{\mathbb{N}} & \cdots& \beta_{3}M_{\mathbb{N}2,\mathbb{N}}\epsilon_{\mathbb{N}} \\ \vdots& \ddots& \vdots\\ \beta_{3}M_{\mathbb{N}2,1}\epsilon_{\mathbb{N}} & \cdots& \beta_{3}M_{\mathbb{N}2,\mathbb{N}}\epsilon_{\mathbb{N}} \end{matrix} \end{matrix} & \begin{matrix} \begin{matrix} \ddots\\ \ldots\end{matrix} & \begin{matrix} \vdots\\ \begin{matrix} \beta_{3}M_{\mathbb{NN},1}\epsilon_{\mathbb{N}} & \cdots& \beta_{3}M_{\mathbb{NN,N}}\epsilon_{\mathbb{N}} \\ \vdots& \ddots& \vdots\\ \beta_{3}M_{\mathbb{NN},1}\epsilon_{\mathbb{N}} & \cdots& \beta_{3}M_{\mathbb{NN,N}}\epsilon_{\mathbb{N}} \end{matrix} \end{matrix} \end{matrix} \end{matrix} \right)_{\mathbb{N}^{2}\times\mathbb{N}^{2}},$$

$$\mathcal{I}_{21}=\left( \begin{matrix} 0 & 0 & \begin{matrix} \ldots& 0 \end{matrix} \\ 0 & 0 & \begin{matrix} \ldots& 0 \end{matrix} \\ \begin{matrix} \vdots\\ 0 \end{matrix} & \begin{matrix} \vdots\\ 0 \end{matrix} & \begin{matrix} \begin{matrix} \ddots\\ \ldots\end{matrix} & \begin{matrix} \vdots\\ 0 \end{matrix} \end{matrix} \end{matrix} \right)_{\mathbb{N}^{2}\times\mathbb{N}^{2}},$$

$$\mathcal{I}_{22}=$$

$$\left( \begin{matrix} \begin{matrix} \beta_{2}L_{11,1}-B_{2} & \cdots& \beta_{2}L_{11,\mathbb{N}} \\ \vdots& \ddots& \vdots\\ \beta_{2}L_{11,1} & \cdots& \beta_{2}L_{11,\mathbb{N}}-B_{2} \end{matrix} & \begin{matrix} \beta_{2}L_{12,1} & \cdots& \beta_{2}L_{12,\mathbb{N}} \\ \vdots& \ddots& \vdots\\ \beta_{2}L_{12,1} & \cdots& \beta_{2}L_{12,\mathbb{N}} \end{matrix} & \begin{matrix} \ldots& \begin{matrix} \beta_{2}L_{1\mathbb{N,}1} & \cdots& \beta_{2}L_{1\mathbb{N,N}} \\ \vdots& \ddots& \vdots\\ \beta_{2}L_{1\mathbb{N,}1} & \cdots& \beta_{2}L_{1\mathbb{N,N}} \end{matrix} \end{matrix} \\ \begin{matrix} \beta_{2}L_{21,1} & \cdots& \beta_{2}L_{21,\mathbb{N}} \\ \vdots& \ddots& \vdots\\ \beta_{2}L_{21,1} & \cdots& \beta_{2}L_{21,\mathbb{N}} \end{matrix} & \begin{matrix} \beta_{2}L_{22,1}-B_{2} & \cdots& \beta_{2}L_{22,\mathbb{N}} \\ \vdots& \ddots& \vdots\\ \beta_{2}L_{22,1} & \cdots& \beta_{2}L_{22,\mathbb{N}}-B_{2} \end{matrix} & \begin{matrix} \ldots& \begin{matrix} \beta_{2}L_{2\mathbb{N,}1} & \cdots& \beta_{2}L_{2\mathbb{N,N}} \\ \vdots& \ddots& \vdots\\ \beta_{2}L_{2\mathbb{N,}1} & \cdots& \beta_{2}L_{2\mathbb{N,N}} \end{matrix} \end{matrix} \\ \begin{matrix} \vdots\\ \begin{matrix} \beta_{2}L_{\mathbb{N}1,1} & \cdots& \beta_{2}L_{\mathbb{N}1,\mathbb{N}} \\ \vdots& \ddots& \vdots\\ \beta_{2}L_{\mathbb{N}1,1} & \cdots& \beta_{2}L_{\mathbb{N}1,\mathbb{N}} \end{matrix} \end{matrix} & \begin{matrix} \vdots\\ \begin{matrix} \beta_{2}L_{\mathbb{N}2,1} & \cdots& \beta_{2}L_{\mathbb{N}2,\mathbb{N}} \\ \vdots& \ddots& \vdots\\ \beta_{2}L_{\mathbb{N}2,1} & \cdots& \beta_{2}L_{\mathbb{N}2,\mathbb{N}} \end{matrix} \end{matrix} & \begin{matrix} \begin{matrix} \ddots\\ \ldots\end{matrix} & \begin{matrix} \vdots\\ \begin{matrix} \beta_{2}L_{\mathbb{NN},1}-B_{2} & \cdots& \beta_{2}L_{\mathbb{NN,N}} \\ \vdots& \ddots& \vdots\\ \beta_{2}L_{\mathbb{NN},1} & \cdots& \beta_{2}L_{\mathbb{NN,N}}-B_{2} \end{matrix} \end{matrix} \end{matrix} \end{matrix} \right)_{\mathbb{N}^{2}\times\mathbb{N}^{2}}$$

This leads to a characteristic equation of degree ${2\mathbb{N}}^{2}$ as follows:

$$\left[ \prod_{i=1}^{\mathbb{N}} \left( x+B_{1}+z_{i} \right)^{\mathbb{N}}\left( 1-\sum_{i=1}^{\mathbb{N}} \frac{\beta_{1}\epsilon_{i}\sum_{m=1}^{\mathbb{N}} K_{iim}}{\left( x+B_{1}+z_{i} \right)} \right) \right]\left[ \left( x+B_{2} \right)^{\mathbb{N}^{2}-1}\left( x+B_{2}(1-\mathcal{R}_{0}^{c}) \right) \right]=0$$

It can be rewritten as

$$\mathcal{A}\left( x \right)\mathcal{B}\left( x \right)=0,$$

where

$$\mathcal{A}\left( x \right)=\left[ \prod_{i=1}^{\mathbb{N}} \left( x+B_{1}+z_{i} \right)^{\mathbb{N}}\left( 1-\sum_{i=1}^{\mathbb{N}} \frac{\beta_{1}\epsilon_{i}\sum_{m=1}^{\mathbb{N}} K_{iim}}{\left( x+B_{1}+z_{i} \right)} \right) \right],$$

$$\mathcal{B}\left( x \right)=\left[ \left( x+B_{2} \right)^{\mathbb{N}^{2}-1}\left( x+B_{2}(1-\mathcal{R}_{0}^{c}) \right) \right].$$

The first case, when $\mathcal{A}\left( x \right)=0$, has the same proof as function $\mathcal{C}_{1}\left( x \right)$ in Theorem 3 with respect to $g_{i}=0$. Hence, $\mathcal{A}\left( x \right)$ has $\mathbb{N}^{2}$ negative solutions that directly satisfy condition (B2). In case two, when $\mathcal{B}\left( x \right)=0$, $\mathcal{B}\left( x \right)$ has $\mathbb{N}^{2}-1$ repeated negative solutions equal to$-B_{2}$ and another negative solution equal to $-B_{2}(1-\mathcal{R}_{0}^{c})$ when $\mathcal{R}_{0}^{c}<1$. The proof is complete.
